# Supplementary material for: Learning-Related Plasticity in Dendrite-Targeting Layer 1 Interneurons
Source: Neuron. 2018 Nov 7;100(3):684–699.e6. doi: 10.1016/j.neuron.2018.09.001 (PMC6226614; doi:10.1016/j.neuron.2018.09.001)
Supplement: Document S1. Figures S1–S7 [file mmc1.pdf]

Neuron, Volume *100*

Supplemental Information

Learning-Related Plasticity in Dendrite-Targeting Layer 1 Interneurons

Elisabeth Abs, Rogier B. Poorthuis, Daniella Apelblat, Karzan Muhammad, M. Belen Pardi, Leona Enke, Dahlia Kushinsky, De-Lin Pu, Max Ferdinand Eizinger, Karl-Klaus Conzelmann, Ivo Spiegel, and Johannes J. Letzkus

Supplemental Figures

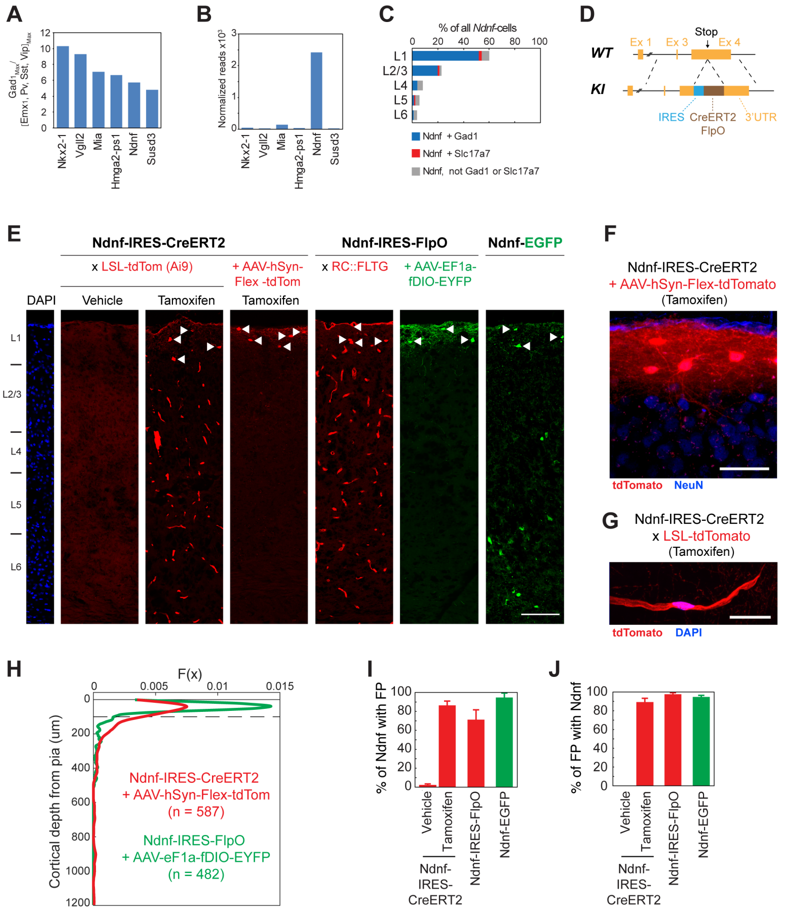

Supplemental Figure 1: Identification of Ndnf as selective marker for L1 INs and generation of mouse lines for selective labeling of* L1 NDNF-INs*. Related to Figure 1

A Enrichment score of the six genes that were identified in the RiboTag-Seq data as highly enriched in Gad2-neurons as compared to excitatory neurons and PV-, SST- and VIP-INs ($Gad2_{Max}/[Emx1/Pv/Sst/Vip]_{Max} \geq 4$). **B** Expression levels of these genes in the Gad2-Cre line as measured by RiboTag-Seq (normalized reads $x10^3$). **C** Quantification of triple FISH for *Ndnf*, *Gad1* and *Slc17a7* (= *VGlut1*) in the adult auditory cortex. Note that *Ndnf*-expressing cells in lower cortical layers are mainly non-neuronal (i.e. *Gad1* and *Slc17a7* negative). **D** Targeting strategy for generation of Ndnf-Ires-CreERT2 and Ndnf-Ires-FlpO mice. A cassette containing Ires-CreERT2 or Ires-FlpO was introduced by homologous recombination into the 3' UTR of the *Ndnf*-gene in Exon 4 after the Stop codon. **E** Reporter expression in Ndnf-Ires-CreERT2 mice, Ndnf-Ires-FlpO mice and in transgenic Ndnf-EGFP mice (Gong et al., 2003). Ndnf-Ires-CreERT2 mice were crossed to mice that express tdTomato upon Cre expression (Ai9, Madisen et al., 2010). Cre activity in the double-heterozygous offspring is observed only upon application of tamoxifen, and reporter expression is observed in L1-INs (arrowheads) as well as in blood vessels throughout all cortical layers (see **G**). Selective labeling of L1 NDNF-

INs (arrowheads) without reporter expression in blood vessels is achieved by injection of Cre-dependent AAVs into the cortex of Ndnf-Ires-CreERT2 mice and subsequent tamoxifen injection (see also **F** for high magnification). Analogous experiments on Ndnf-Ires-FlpO mice. Ndnf-Ires-FlpO mice were crossed to mice that express tdTomato upon Flp expression (RC::FLTG), revealing expression in both blood vessels and L1-INs (arrowheads). Injection of Flp-dependent AAVs into the cortex of these mice labels selectively L1 NDNF-INs (arrowheads). Ndnf-EGFP labels L1 NDNF-INs (arrowheads) and PNs in lower cortical layers (no labeling of PNs in any layers was observed in Ndnf-Ires-CreERT2 mice) (scale-bar in **E** 200 µm, in **F, G** 20 µm). **H** Distribution of tdTomato- or EYFP-labeled cells in Ndnf-Ires-CreERT2 and Ndnf-Ires-FlpO mice injected with conditional AAVs (AAV-hSyn-Flex-tdTomato or AAV-EF1a-fDIO-EYFP). Labeled cells are highly concentrated in L1 in both lines. **I, J** *Ndnf*-expression in L1 is accurately reported by Ndnf-Ires-CreERT2, Ndnf-Ires-FlpO and Ndnf-EGFP mice. FISH for *Ndnf* and the respective fluorescent protein (*tdTomato* or *Egfp)* was done in adult brains of either double heterozygous Ndnf-Ires-CreERT2 x Ai9 mice or of Ndnf-EGFP mice. The percentages of co-expressing cells in L1 of the auditory cortex are plotted in the bar graphs. Error bars are s.e.m.

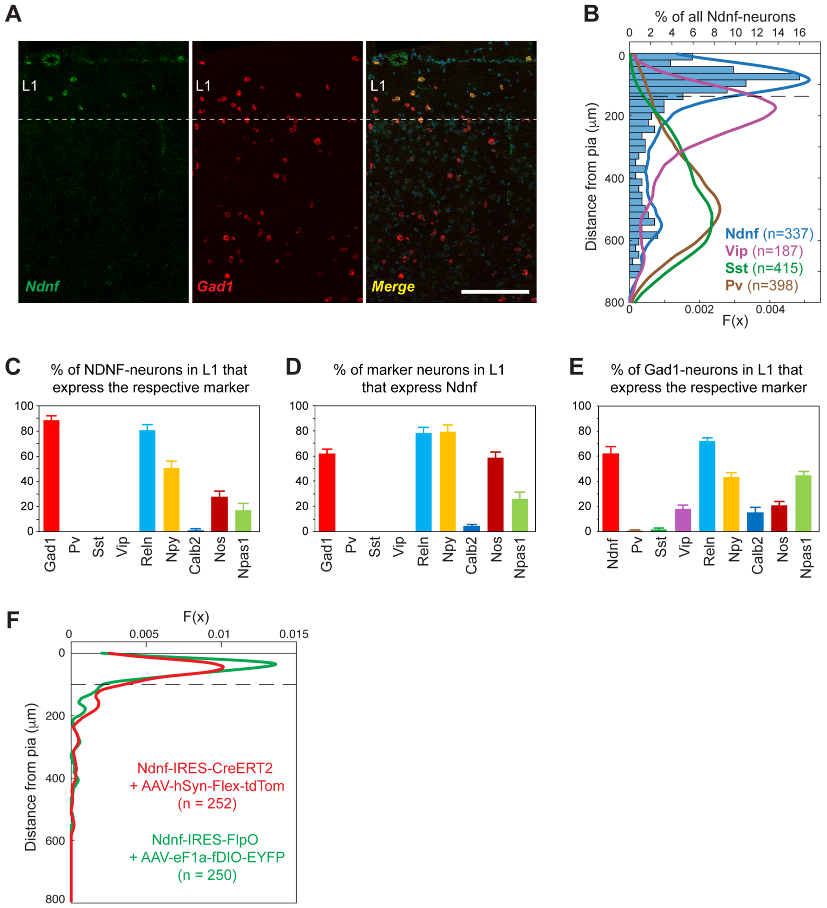

Supplemental Figure 2: Ndnf as selective marker for L1 INs in prefrontal cortex and targeting by novel mouse lines. Related to Figure 1

A Representative image of FISH for *Ndnf* and *Gad1* in adult prefrontal cortex (DAPI-labeled nuclei are in blue, scale-bar = 200 µm). **B** The distribution of *Ndnf*-expressing INs differs from the distribution of other INs in prefrontal cortex. The distance from the pia was determined for each cell expressing a given marker, and plotted as a histogram (for *Ndnf*) or the corresponding Probability Density Function (PDF, for *Ndnf*, *Pv*, *Sst* and *Vip*, dashed line indicates the L1 border). **C-F** *Ndnf*-expressing neurons constitute the majority of L1 GABAergic neurons and do not overlap with *Pv*, *Sst* or *Vip*. **C** Percentage of L1 *Ndnf* neurons that co-express the respective marker. **D** Percentage of L1 neurons that express the respective subtype marker and that co-express *Ndnf*. **E** Percentage of Gad1-positive L1 neurons that express each of the subtype markers. **F** Two newly generated mouse lines allow for selective labeling of L1 NDNF neurons in prefrontal cortex. Distribution of tdTomato- or EYFP-labeled cells in Ndnf-Ires-CreERT2 and Ndnf-Ires-FlpO mice injected with an conditional AAVs (AAV-hSyn-Flex-tdTomato or AAV-EF1a-fDIO-EYFP, respectively). Labeled cells are highly concentrated in L1 in both lines. Data are presented as mean ± s.e.m.

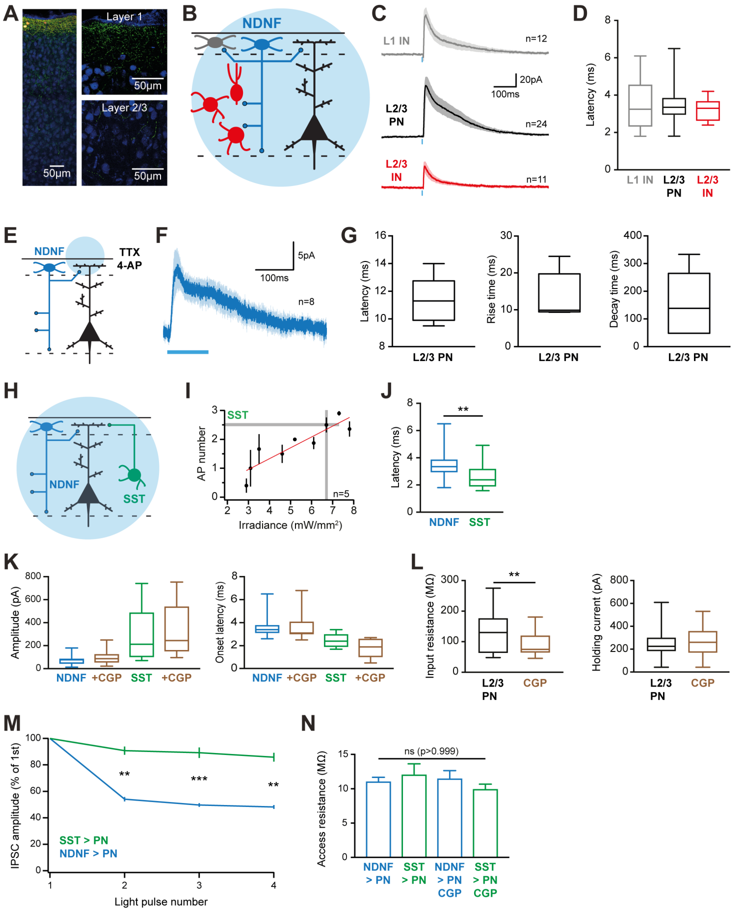

Supplemental Figure 3: Further characterization of output connectivity of layer 1 NDNF interneurons in auditory cortex. Related to Figure 2

A Overview (**left**) and high magnification images (**right**) of L1 NDNF-IN output synapse location after AAV-mediated expression of cytosolic tdTomato (red) and synaptophysin-GFP fusion protein (green) in auditory cortex of Ndnf-Ires-CreERT2 mouse line counterstained for NeuN (blue). Note the higher density of GFP puncta in L1 compared to L2/3 (images on right are projections over 2 µm imaging depth). **B, C** Schematic of optogenetic mapping and average traces of optogenetically-evoked IPSCs in CHR-2 negative L1-INs (grey, n=12), L2/3 PNs (black, n=24) and L2/3 INs (red, n=11). **D** Optogenetically-evoked IPSCs showed short onset latencies that did not differ between the postsynaptic populations (Kruskal-Wallis H-test with Dunn's multiple comparison). **E, F** Schematic and average IPSC in response to optogenetic activation of L1 NDNF-IN synapses selectively in L1 under action potential block (TTX, 1 µM, 4-AP 100 µM). **G** Latencies, rise and decay times of IPSCs in these experiments (n=8). **H** Schematic of experiments comparing the properties of NDNF- and SST-IN input to the distal dendrites of L2/3 PNs. **I** Calibration of optogenetic stimulation of ChR-2 expressing SST-INs. Across the population (n=5), the chosen irradiance (grey lines, 6.8 mW/mm^2) elicited 2.5 action potentials per pulse (0.5 ms). **J** The onset latency of L1 NDNF-IN input to

L2/3 PNs was significantly longer than for SST input, consistent with the slower rise and decay times of this input (**Figure 2I**, Mann-Whitney test). **K** Bath application of the selective $GABA_B$ receptor antagonist CGP 55845 (3 μM) caused no significant change in amplitude or onset latency of IPSCs from L1 NDNF-INs (n=9) or SST-INs (n=7, Wilcoxon test within NDNF and SST groups). **L** CGP 55845 (3 μM) caused only modest effects on the intrinsic properties of L2/3 PNs (n=16, Wilcoxon test). **M** Short-term dynamics of SST- and NDNF-IN input to PNs during optogenetic train stimulation normalized to the amplitude of the first IPSC. Note stronger short-term depression of L1 NDNF-IN output synapse, consistent with previous observations during paired recordings (Capogna and Pearce, 2011; Olah et al., 2009; Overstreet-Wadiche and McBain, 2015; Tamas et al., 2003). **N** Maximal access resistance of recordings from L2/3 PNs during input from L1 NDNF-INs (n=24) or SST-INs (n=13), and of the subset of these recordings in which the $GABA_B$ receptor antagonist CGP 55845 was applied (L1 NDNF-INs n=9, SST-INs n=7). There is no difference in access resistance between these groups (Kruskal-Wallis H-test with Dunn's multiple comparison, p>0.999), indicating that recording quality cannot explain the physiological differences we observe. C, F, I, N show mean ± s.e.m., other plots range, quartiles and median.

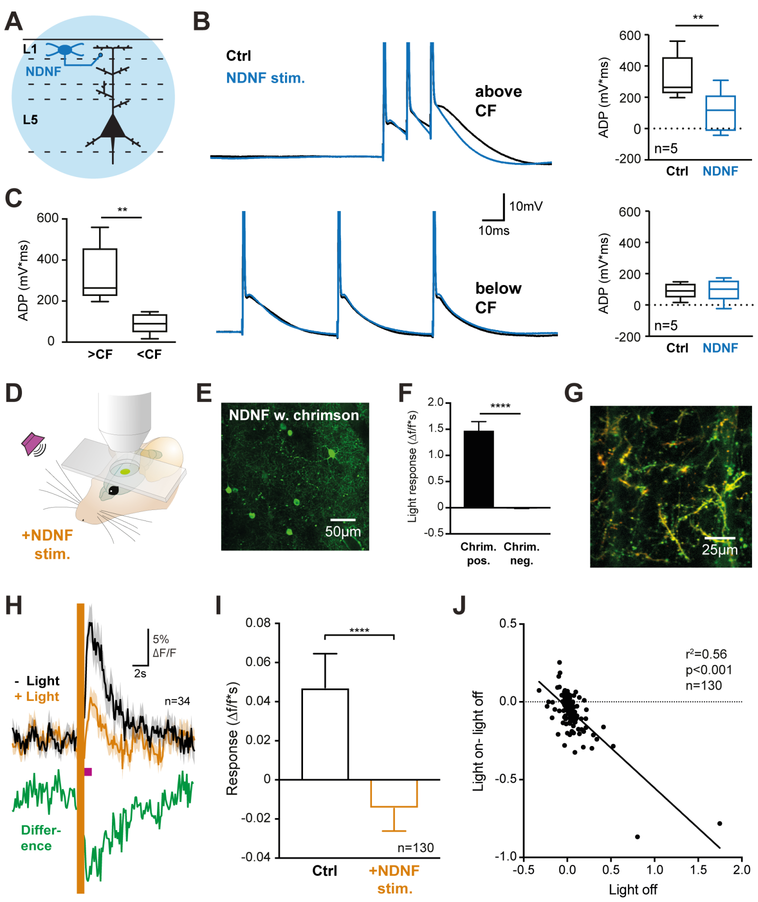

Supplemental Figure 4: Further characterization of pyramidal neuron dendritic activity control by layer 1 NDNF interneurons. Related to Figure 3

A Schematic of critical frequency experiment in L5 PNs. **B** Example recording (left) and quantification (right, n=5) showing the selective reduction of the afterdepolarization (ADP) after an action potential burst by preceding L1 NDNF-IN stimulation (4 pulses at 40Hz, ending 50-100 ms before last action potential) for supra- but not sub-critical frequency stimulation (paired t-test). **C** The ADP after a burst of 3 action potentials was greater for supra- than for sub-critical frequency stimulation (n=9, paired t-test). **D** *In vivo* 2-photon imaging in auditory cortex of awake mice combined with sensory stimulation (magenta, 5 white noise bursts, 100 ms duration, delivered at 5 Hz) and optogenetic activation of L1 NDNF-INs (yellow, 594 nm). **E** Field of view during *in vivo* imaging of L1 NDNF-INs co-expressing GCaMP6s (green) and the optogenetic effector Chrimson in Ndnf-Ires-FlpO mice. **F** Optogenetic activation elicited strong responses in L1 NDNF-INs expressing Chrimson (n=78), and no activity in animals that only expressed GCaMP6s (n=134, Mann-Whitney test). **G** Field of view during *in vivo* imaging of distal PN dendrites in L1 expressing GCaMP6s (green) and tdTomato (red) used for motion correction. PNs were selectively labeled by a combination of retrograde Cre-expression from subcortical regions and Cre-dependent expression of GCaMP6s and tdTomato in auditory cortex. **H** Sensory responses (black) in dendritic branches that displayed

significant activation by auditory stimulation (34 dendrites in 3 mice, see Methods for details). Optogenetic activation of L1 NDNF-INs (yellow) immediately preceding auditory stimulation (magenta) caused a long lasting reduction of dendritic responses (difference trace in green). **I** There was a highly significant reduction in dendritic responses also when both dendrites responsive and unresponsive to sensory stimulation were considered (130 dendrites in 3 mice, Wilcoxon test, see Methods for details). **J** There was a highly significant correlation between the size of sensory responses in a dendrite (light off) and the effect of L1 NDNF-IN activation (light on – light off, r^2=0.56, p<0.001, n=130), revealing that stronger sensory responses were suppressed more. F, H, I shows mean ± s.e.m., other plots range, quartiles and median.

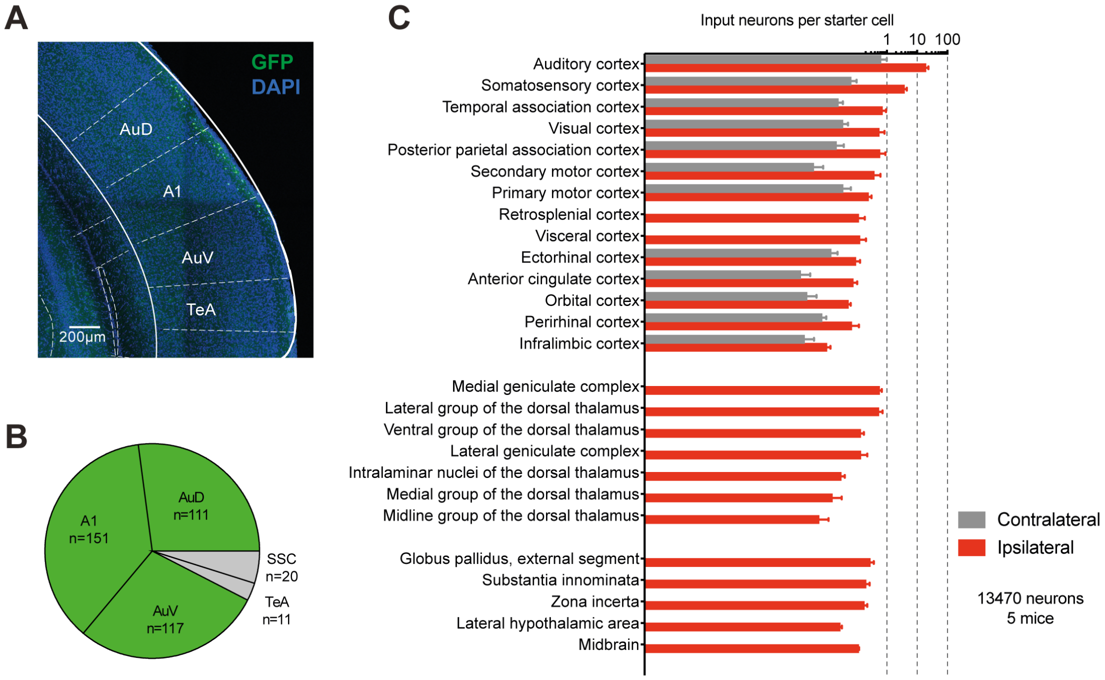

Supplemental Figure 5: Further characterization brain-wide sources of synaptic input to auditory cortex layer 1 NDNF interneurons. Related to Figure 4

A Representative image after injection of AAV-synP-DIO-sTpEpB (Kohara et al., 2014) to make L1 NDNF-INs competent for transduction by rabies virus. Note expression in auditory cortex layer 1. The mCherry channel revealing presynaptic input neurons is omitted here for clarity, see **Figure 4A** for a two color image. **B** Area localization of all starter L1 NDNF-INs identified by GFP and mCherry co-localization (410 cells, 5 mice). 92% of starter cells were located in auditory cortex (A1 primary auditory cortex, AuD dorsal auditory cortex, AuV ventral auditory cortex). An additional 3% was located in temporal association cortex (TeA), whereas 5% were found in adjacent somatosensory cortex (SSC). **C** The same data as in **Figure 4C** is presented here by the complementary measure of number of input neurons per starter cell. Plot shows mean ± s.e.m.

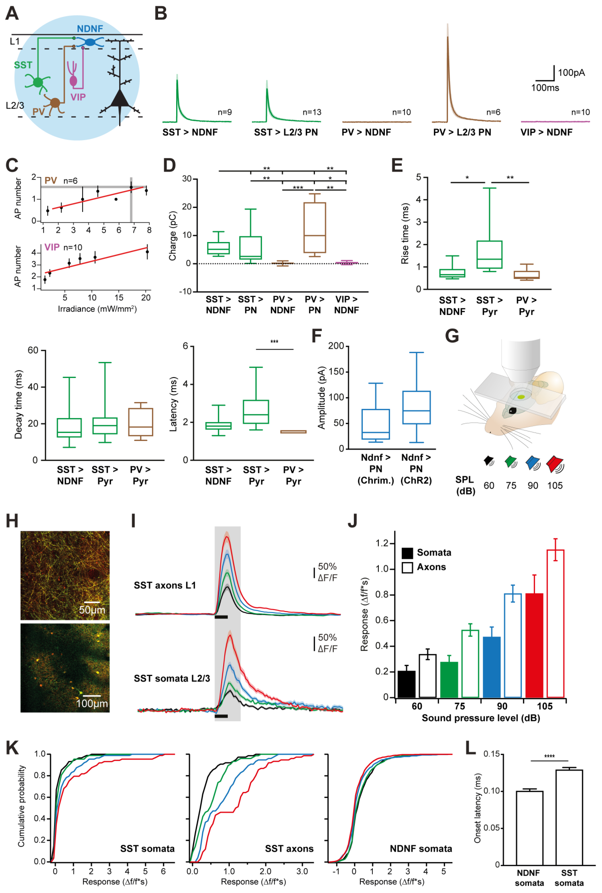

Supplemental Figure 6: Further characterization of inhibitory control of layer 1 NDNF interneuron activity in auditory cortex. Related to Figure 5

A, B Schematic and average traces of optogenetically-evoked IPSCs in L1 NDNF-INs (from SST n=9, from PV n=10, from VIP n=10) and L2/3 PNs recorded for comparison (from SST n=13, from PV n=6). The connection from VIP-INs to PNs was not tested due to the weak connectivity observed in previous studies (Pfeffer et al., 2013; Pi et al., 2013). **C** Calibration of optogenetic stimulation of ChR-2 expressing PV- and VIP-INs. In PV-INs (n=6), the chosen irradiance (grey lines, 6.8 mW/mm^2) elicited 1.6 action potentials per pulse (0.5 ms). Connections from VIP-INs were tested at different irradiance levels between 5 and 20 W/mm^2, which elicit robust action potential firing (n=10). **D** L1 NDNF-INs receive strong inhibition from SST-INs similar to L2/3 PNs, but no input from PV- or VIP-INs (Kruskal-Wallis H-test with Dunn's multiple comparison). **E** While decay times were similar in the different experiments, rise times of SST input to L2/3 PNs were longer than in the other populations, consistent with the distal dendritic location of these synapses. In addition, the onset latencies of PV-IN input to PNs was faster than in the other populations, consistent with the perisomatic localization and rapid kinetics of these synapses (Kruskal-Wallis H-test with Dunn's multiple comparison). **F** Comparison of IPSC amplitudes elicited in PNs by optogenetic stimulation after Cre-dependent expression of ChR-2 and Flp-dependent expression of Chrimson. No

difference was observed (p>0.05, unpaired t-test), indicating similar efficiency of L1 NDNF-IN stimulation in both experiments. **G** Schematic for *in vivo* 2-photon imaging in auditory cortex of awake mice during stimulation with white noise bursts (5 bursts, 100 ms duration, delivered at 5 Hz) at different sound pressure levels. **H** Fields of view during *in vivo* imaging of SST axons in L1 (**top**), SST-INs in L2/3 (**bottom**) in auditory cortex. **I** SST-IN axons (**top**, n=11 regions in 11 mice) and somata (**bottom**, n=88 in 5 mice) displayed similar response increases with increasing stimulus intensity (60-105 dB SPL, color code in **G**). **J** Statistics of responses in **I** (Kruskal-Wallis test with Dunn's multiple comparison, only significant differences are reported, somata: s, axons: a: s 60dB vs s 90dB: p<0.05, s 60dB vs s 105dB: p<0.0001, s 60dB vs a 60dB: p<0.01, s 60dB vs a 75dB: p<0.0001, s 60dB vs a 90dB: p<0.0001, s 60dB vs a 105dB: p<0.0001, s 75dB vs s 105dB: p<0.05, s 75dB vs a 75dB: p<0.0001, s 75dB vs a 75dB: p<0.0001, s 75dB vs a 90dB: p<0.0001, s 75dB vs a 105dB: p<0.0001, s 90dB vs a 90dB: p<0.0001, s 90dB vs a 105dB: p<0.0001, s 105dB vs a 90dB: p<0.001, s 105dB vs a 90dB: p<0.001, s 105dB vs a 105dB: p<0.0001, a 60dB vs a 90dB: p<0.0001). This indicates that both approaches yield comparable results, and that imaging SST axons is a valid approach to determine the inhibition that arrives in L1 from this source (c.f. Lovett-Barron et al., 2014). **K** Cumulative histograms of response integrals of SST somata in L2/3 (left, 88 neurons per SPL, 5 mice), SST axons in L1 (center, 77 responses per SPL, 11 regions in 11 mice) and L1 NDNF-INs (right, 95 neurons per SPL, 5 mice). Note opposite effects of stimulus intensity across the entire population. **L** Latencies of the initial excitatory component in L1 NDNF-INs and SST axons. Note faster onset of L1 NDNF-IN excitation (Mann-Whitney test). B, C, I, J, L show mean ± s.e.m., other plots range, quartiles and median.

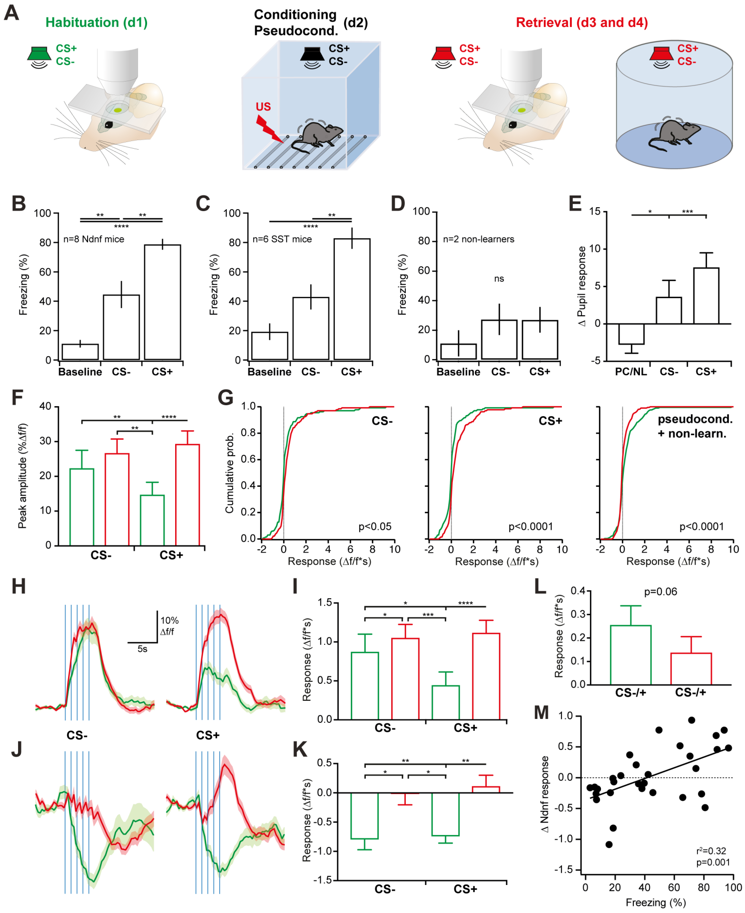

Supplemental Figure 7: Further characterization of plasticity of layer 1 NDNF interneuron stimulus responses after associative learning. Related to Figure 6

A Experimental schematic. **B, C** Freezing behavior of fear conditioned NDNF (**B**, n=8, CS+: 4 up sweeps, 4 down sweeps) and SST animals (**C**, n=6, CS+: 4 up sweeps, 2 down sweeps) in a freely-behaving memory retrieval session on day 3 or 4 indicates strong, discriminative fear memory (one-way ANOVA NDNF $F(2, 21)=32.7$, $p<0.0001$; one-way ANOVA SST $F(2, 15)=19.8$, $p<0.0001$ Tukey's multiple comparison test). **D** Freezing behavior of 2 fear conditioned NDNF animals which failed to form a stable memory (criterion: CS+ freezing < 40%, one-way ANOVA NDNF $F(2, 3)=0.97$, $p>0.05$). **E** Change in CS-evoked pupil responses between the first and second imaging session for pseudoconditioning and non-learners (CS1 and 2 combined, 7 mice) and fear conditioning (8 mice, One-way ANOVA $F(2, 27)=12.2$, $p<0.001$, Tukey's posthoc test). **F** Similar to the response integral (**Figure 6H**), the peak amplitudes of L1 NDNF-IN responses were similar during habituation ($p=0.85$, green), and strongly increased for the CS+ during retrieval (red), with a modest increase also observed for the CS- (133 neurons in 8 mice, CS+: 58 neurons up sweeps, 75 neurons down sweeps, Friedman test with Dunn's multiple comparison). **G** Cumulative histograms of response integrals for CS-, CS+ and pseudoconditioned stimuli (pseudoconditioned and non-learners: 128 neurons in 7 mice, both CSs combined). Note the potentiation of CS+ responses across

the population (Wilcoxon test). **H** Average timecourse of CS responses of strongly activated L1 NDNF-INs (see Methods, CS+ 69 neurons, CS- 68 neurons) showing a modest increase for the CS-, and strong potentiation of CS+ responses (onset of each sweep marked by blue lines). **I** Quantification of the response integral for data in **H** (Friedman test with Dunn's multiple comparison). Both CS- and CS+ responses were significantly potentiated after fear conditioning, whereas no difference was observed during habituation. **J** Same as **H** for strongly inhibited L1 NDNF-INs (see Methods, CS+: 20 neurons, CS-: 16 neurons, Friedman test with Dunn's multiple comparison). Inhibitory CS responses are reduced for the CS-, and converted to delayed excitation for the CS+. **K** Quantification of the response integral for data in **J**. **L** Response integral from 2 fear conditioned NDNF animals which failed to form a stable memory (n=44, Wilcoxon test). Since no difference in behavior was observed (**D**), responses to both CSs are presented combined. **M** Correlation between the response change in L1 NDNF-INs due to fear conditioning (response integral retrieval minus integral habituation) and freezing behavior elicited by that stimulus for fear conditioned and pseudoconditioned animals indicates that potentiation of L1 NDNF-INs correlates with learned stimulus relevance. Data are presented as mean ± s.e.m.
