# Supplementary material for: Learning-Related Plasticity in Dendrite-Targeting Layer 1 Interneurons
Source: Neuron. 2018 Nov 7;100(3):684–699.e6. doi: 10.1016/j.neuron.2018.09.001 (PMC6226614; doi:10.1016/j.neuron.2018.09.001)
Supplement: Document S2. Article plus Supplemental Information [file mmc2.pdf]

Neuron

Article

Learning-Related Plasticity in Dendrite-Targeting Layer 1 Interneurons

Highlights

- NDNF is a selective marker for neocortical layer 1 interneurons
- NDNF interneurons mediate prolonged inhibition of distal pyramidal neuron dendrites
- Inhibition from Martinotti cells tightly controls NDNF interneuron responses
- Dendritic inhibition by NDNF interneurons is highly experience dependent

Authors

Elisabeth Abs, Rogier B. Poorthuis, Daniella Apelblat, ..., Karl-Klaus Conzelmann, Ivo Spiegel, Johannes J. Letzkus

Correspondence

ivo.spiegel@weizmann.ac.il (I.S.), johannes.letzkus@brain.mpg.de (J.J.L.)

In Brief

Using a selective marker for neocortical layer 1 interneurons, Abs, Poorthuis, et al. identify these little-understood cells as a powerful, highly experience-dependent source of inhibition in pyramidal neuron dendrites that is in turn controlled by activity in the local circuit.

Abs et al., 2018, Neuron *100*, 684–699
November 7, 2018 © 2018 The Authors. Published by Elsevier Inc.
https://doi.org/10.1016/j.neuron.2018.09.001

CellPress

Neuron
Article

Learning-Related Plasticity in Dendrite-Targeting Layer 1 Interneurons

Elisabeth Abs,[1,4] Rogier B. Poorthuis,[1,4] Daniella Apelblat,[2] Karzan Muhammad,[1] M. Belen Pardi,[1] Leona Enke,[1] Dahlia Kushinsky,[2] De-Lin Pu,[1] Max Ferdinand Eizinger,[3] Karl-Klaus Conzelmann,[3] Ivo Spiegel,[2,5,*] and Johannes J. Letzkus[1,5,6,*]
[1]Max Planck Institute for Brain Research, 60438 Frankfurt, Germany
[2]Department of Neurobiology, Weizmann Institute of Science, 76100 Rehovot, Israel
[3]Max von Pettenkofer Institute, Virology, Medical Faculty and Gene Center, Ludwig Maximilians University, 81377 Munich, Germany
[4]These authors contributed equally
[5]Senior author
[6]Lead Contact
*Correspondence: ivo.spiegel@weizmann.ac.il (I.S.), johannes.letzkus@brain.mpg.de (J.J.L.)
https://doi.org/10.1016/j.neuron.2018.09.001

SUMMARY

A wealth of data has elucidated the mechanisms by which sensory inputs are encoded in the neocortex, but how these processes are regulated by the behavioral relevance of sensory information is less understood. Here, we focus on neocortical layer 1 (L1), a key location for processing of such top-down information. Using Neuron-Derived Neurotrophic Factor (NDNF) as a selective marker of L1 interneurons (INs) and *in vivo* 2-photon calcium imaging, electrophysiology, viral tracing, optogenetics, and associative memory, we find that L1 NDNF-INs mediate a prolonged form of inhibition in distal pyramidal neuron dendrites that correlates with the strength of the memory trace. Conversely, inhibition from Martinotti cells remains unchanged after conditioning but in turn tightly controls sensory responses in NDNF-INs. These results define a genetically addressable form of dendritic inhibition that is highly experience dependent and indicate that in addition to disinhibition, salient stimuli are encoded at elevated levels of distal dendritic inhibition.

INTRODUCTION

Layer 1 (L1) is a unique site in the neocortex. It is immediately recognizable, as it contains relatively few somata and is instead comprised primarily of the apical dendrites of local pyramidal neurons (PNs) and a number of long-range projections that convey contextual, top-down information (Douglas and Martin, 2004; Felleman and Van Essen, 1991). It has therefore been suggested that L1 is a key site where information about the behavioral relevance of a stimulus is received and integrated with the representation of its bottom-up attributes in lower layers (Cauller, 1995; Larkum, 2013). This process is thought to occur in the distal dendrites of PNs, which show highly specialized information processing capacities such as regenerative events called dendritic spikes (Helmchen et al., 1999; Major et al., 2013; Stuart and Spruston, 2015). In line, recent *in vivo* studies have highlighted the important role of dendritic spikes during sensorimotor integration, motor learning, and perception (Cichon and Gan, 2015; Takahashi et al., 2016; Xu et al., 2012).

In turn, dendritic computations are powerfully controlled by inhibition (Major et al., 2013; Stuart and Spruston, 2015). The best-understood source of distal dendritic inhibition in L1 originates from projections of somatostatin (SST)-positive Martinotti cells located in deeper layers (Higley, 2014; Yavorska and Wehr, 2016). SST-interneurons (INs) receive little thalamic input but instead are strongly driven by recurrent excitation from the local PNs and are therefore thought to provide dendritic inhibition that is proportional to the ongoing activity in the PN network (Adesnik et al., 2012; Yavorska and Wehr, 2016). A second source of inhibition in L1 that is far less understood derives from the sparse set of L1-INs. These cells receive input from a range of top-down projections, including the cholinergic system, higher-order thalamus, and cortico-cortical feedback in rodents (Bennett et al., 2012; Cruikshank et al., 2012; Letzkus et al., 2011; Palmer et al., 2012; Zhu and Zhu, 2004), and recent data have revealed strong cholinergic responses in L1 INs also in the human neocortex (Poorthuis et al., 2018). This suggests that, like distal dendritic excitation, inhibition from L1 INs may also be governed by internally generated activity representing, for instance, the behavioral relevance of sensory information (Letzkus et al., 2015). However, while slice recordings have defined two major types of L1 INs (elongated neurogliaform cells [eNGCs] and single-bouquet cell-like neurons [SBCs]; Chu et al., 2003; Jiang et al., 2013, 2015; Letzkus et al., 2011, 2015; Palmer et al., 2012; Wozny and Williams, 2011), the investigation of L1-INs has been hampered by the lack of selective genetic access that is key for a multidisciplinary understanding of the circuit and behavioral function of these INs (Kepecs and Fishell, 2014; Letzkus et al., 2015; Lovett-Barron and Losonczy, 2014; Wester and McBain, 2014).

Here, we establish Neuron-Derived Neurotrophic Factor (NDNF; Kuang et al., 2010) as a highly selective marker for L1

This is an open access article under the CC BY license (http://creativecommons.org/licenses/by/4.0/).

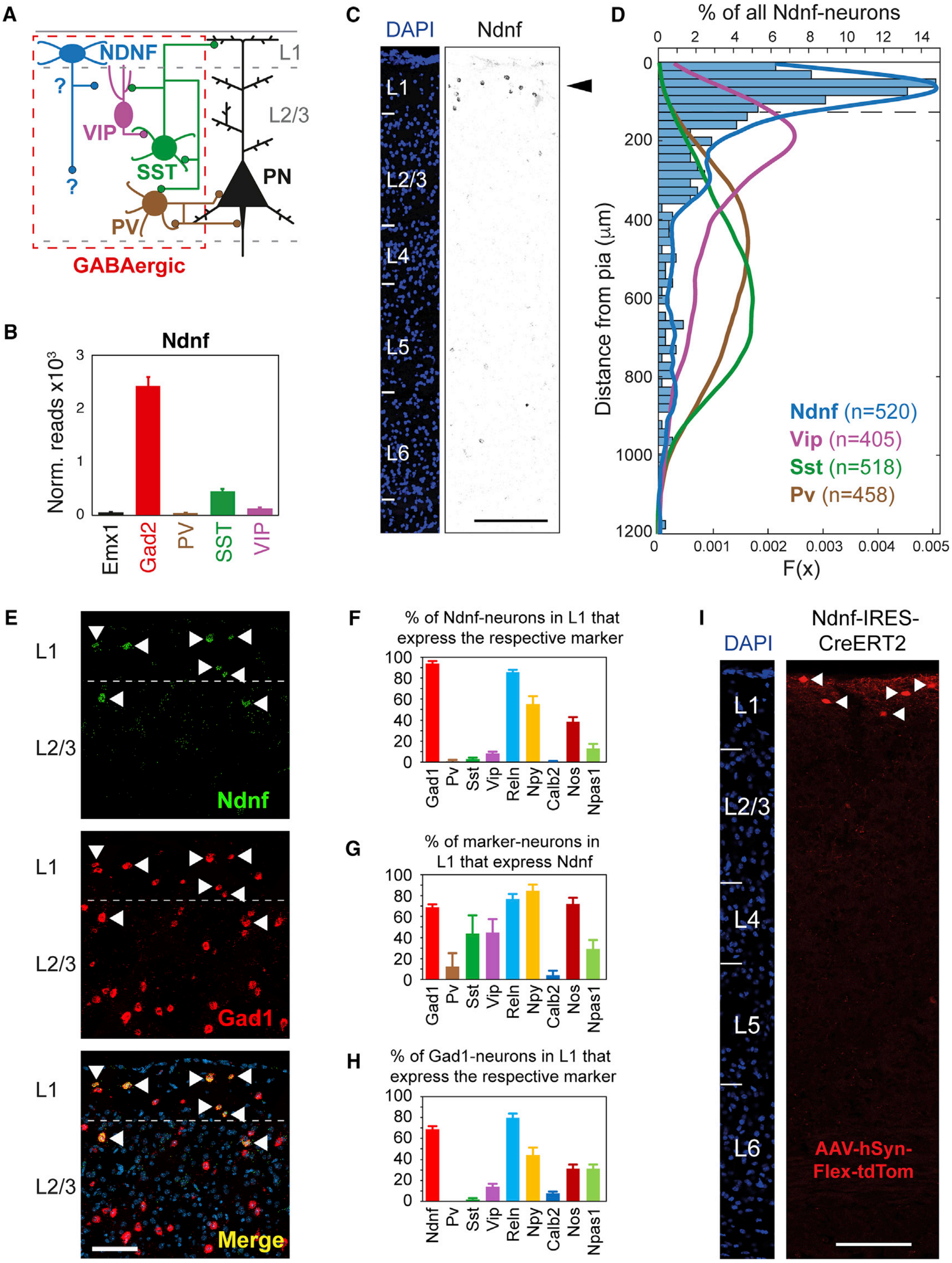

Figure 1. Ndnf Is a Selective Marker of Neocortical Layer 1 Interneurons

(A) Circuit diagram of neocortex. The connections of L1 NDNF-INs are not known and are the subject of this study.

(B) RiboTag-seq indicates that *Ndnf*-expression is highly enriched in GABAergic neurons, but not in IN subtypes that express *Pv*, *Sst*, or *Vip*.

(C and D) *Ndnf*-expressing GABAergic neurons are concentrated in L1. RNAscope FISH for *Ndnf*, *Gad1*, and IN subtype markers was done in the adult auditory cortex.

(C) *Ndnf*-expressing cells are concentrated in L1 (scale bar, 200 μm).

(legend continued on next page)

INs in the adult auditory and prefrontal cortex and generate a tamoxifen-inducible Cre allele as well as a Flp allele to specifically target these neurons. We report data on the molecular properties and the synaptic input and output organization of auditory cortex L1 NDNF-INs, with a focus on their prominent interaction with SST-INs both *in vitro* and *in vivo*. In addition, to induce auditory cortex plasticity, we employ associative fear conditioning (LeDoux, 2000; Weinberger, 2007) to identify and characterize plastic changes of L1 NDNF and SST-IN responses during expression of behavioral memory.

RESULTS

NDNF Is a Selective Marker of Neocortical L1 INs

To identify a selective marker for L1-INs, we used a subtractive approach on RNA sequencing (RNA-seq) data generated by sequencing of ribosome-bound RNAs purified from genetically defined subtypes of cortical neurons that express a Cre-dependent epitope tagged ribosomal protein (i.e., RiboTag; Mardinly et al., 2016; Sanz et al., 2009). We reasoned that a marker of L1 GABAergic INs must be highly enriched in the RNAs isolated from Gad2-Cre mice (Taniguchi et al., 2011) as compared to RNAs purified from cortical excitatory neurons (Emx1-Cre mice; Gorski et al., 2002) and from three nonoverlapping subtypes of INs that together account for ~85% of all INs and are largely absent from L1 (Figure 1D, SST, parvalbumin [PV], and vasoactive intestinal polypeptide [VIP] neurons, labeled by the respective Cre lines; Hippenmeyer et al., 2005; Taniguchi et al., 2011). Indeed, a transcriptome-wide search identified six genes that are substantially enriched in Gad2-derived RNAs compared to all the other RNAs (i.e., $Gad2_{Max}/[Emx1/Pv/Sst/Vip]_{Max} \geq 4$; Figure S1A). Out of these candidates *Ndnf* is the only gene that is expressed at appreciable levels (Figures S1B and S1C), making it a promising candidate marker for L1 INs.

Consistent with this, fluorescent *in situ* hybridization (FISH) indicated that *Ndnf*-expressing cells are highly concentrated in L1 in an expression pattern that is very different from the well-described IN subtypes (Figures 1C and 1D). Direct comparison to pan-GABAergic and pan-glutamatergic markers further revealed that nearly all *Ndnf* cells are GABAergic and that the few *Ndnf* cells that are Gad1 negative are likely non-neuronal, as most of them also do not express VGlut1 (Figure S1C). Together, these data indicate that *Ndnf* is a specific marker of L1 INs in the adult cortex. These findings are consistent with recent studies that used single-cell RNA-seq to analyze the molecular composition of cortical neurons (Cadwell et al., 2016; Habib et al., 2017; Tasic et al., 2016; Zeisel et al., 2015). We next used triple FISH to directly compare the expression of *Ndnf* and *Gad1* with multiple markers of (L1) IN subtypes emerging from these studies (Figures 1E–1H). We find that *Ndnf* is expressed in roughly two-thirds of all L1 INs and that L1 NDNF-INs do not overlap with the IN populations defined by *Pv*, *Sst*, *Vip*, or *Calb2* (Calretinin). In contrast, expression of *Ndnf* showed large overlap with *Reln* (Reelin) and *Npy* (Neuropeptide Y) and slightly weaker colocalization with *nNos*, markers that, unlike *Ndnf*, are also expressed widely in lower cortical layers and are thus less suitable for selective identification of L1 INs. Taken together, these data demonstrate that out of the genes analyzed, *Ndnf* is by far the most selective marker for L1 INs. In addition, co-expression of *Reln, Npy*, and *nNos* suggests that L1 NDNF-INs may correspond to eNGCs (see Discussion; Overstreet-Wadiche and McBain, 2015; Tasic et al., 2016). This is further supported by the low co-expression of *Ndnf* with *Npas1* (Figures 1F–1H), which has been reported to be absent from eNGCs but expressed in L1 SBCs (Cadwell et al., 2016; Habib et al., 2017).

Based on these findings, we generated a knockin mouse allele in which a tamoxifen-inducible version of Cre recombinase is expressed under the control of the endogenous *Ndnf* locus (Ndnf-Ires-CreERT2; Figure S1D). When crossed to a reporter strain (Ai9; Madisen et al., 2010), this allele drives reporter expression only in the presence of tamoxifen (Figure S1E), indicating tight temporal control of Cre activity and thereby differing from previously generated NDNF-Cre mice (Tasic et al., 2016). Similar to endogenous *Ndnf* expression (Figures 1C and 1D), tdTomato-labeled neurons are found primarily in L1 (Figure S1E), and nearly all of these tdTomato-labeled L1 INs co-express endogenous *Ndnf* (Figures S1I and S1J). Similar to previous results (Tasic et al., 2016), this line also labels blood vessels throughout the cortex when used in conjunction with Ai9 reporter mice (Figures S1E and S1G). Importantly, when used in combination with conditional adeno-associated viral vector (AAV) constructs our knockin allele labels very selectively only L1 NDNF-INs (Figures 1I, S1E, S1F, and S1H). To further extend our experimental capabilities, we went on to generate a second knockin mouse allele in which Flp recombinase is expressed under the control of the endogenous *Ndnf* locus (Ndnf-Ires-FlpO; Figure S1D). This line displayed similar selectivity for L1 (Figures S1E and S1H) and similar selectivity and fidelity for labeling *Ndnf*-positive neurons as the Cre line (Figures S1I and S1J), indicating that both mouse alleles are powerful tools for circuit dissection. Moreover, we addressed the specificity of a

(D) The distribution of *Ndnf*-expressing INs in the adult auditory cortex differs from the distribution of other INs. The distance from the pia was determined for each cell expressing a given marker and plotted as a histogram (for *Ndnf*) or the corresponding probability density function (PDF; for *Ndnf*, *Pv*, *Sst*, and *Vip*; dashed line indicates the L1 border).

(E–H) *Ndnf*-expressing neurons constitute the majority of L1 GABAergic neurons and do not overlap with *Pv*, *Sst*, or *Vip*.

(E) Representative image of FISH for *Ndnf* and *Gad1* in the auditory cortex (co-expressing neurons are indicated by arrowheads, and DAPI-labeled nuclei are in blue; scale bar represents 100 μm).

(F) Percentage of L1 *Ndnf* neurons that co-express the respective marker.

(G) Percentage of L1 neurons expressing the respective subtype marker that co-expresses *Ndnf*.

(H) Percentage of Gad1-positive L1 neurons expressing each subtype marker.

(I) A newly generated mouse line allows for temporally controlled selective labeling of L1 NDNF-neurons. Auditory cortex section of an Ndnf-Ires-CreERT2 mouse injected with an AAV-construct that drives Cre-dependent tdTomato expression (AAV-hSyn-Flex-tdTomato) upon tamoxifen application (scale bar, 200 μm). Data are presented as mean ± SEM.

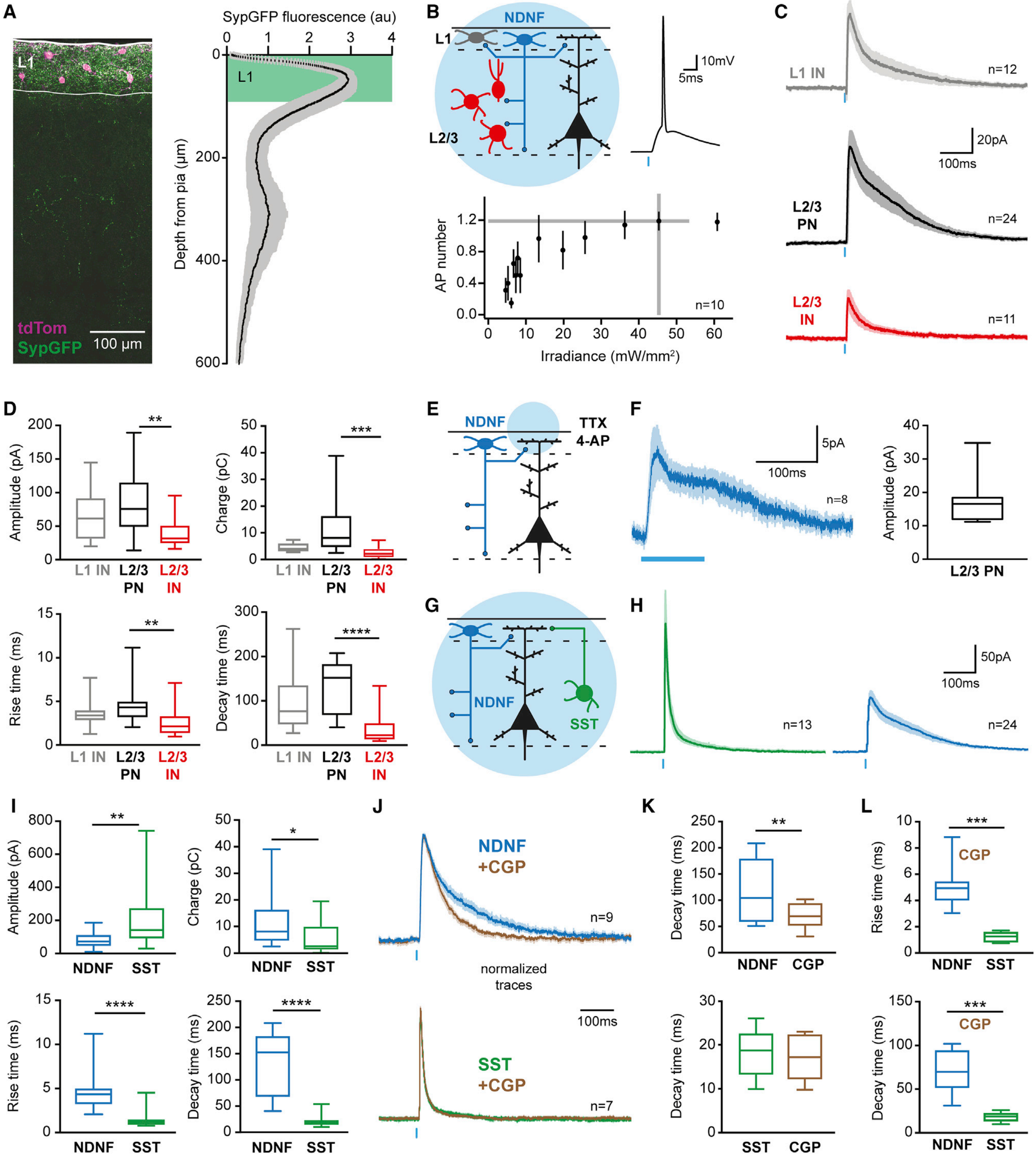

Figure 2. Output Connectivity of Layer 1 NDNF-Interneurons in the Auditory Cortex

(A) AAV-mediated expression of tdTomato and synaptophysin-GFP in the Ndnf-Ires-CreERT2 mouse auditory cortex (left). Synaptophysin-GFP fluorescence is strongly enriched in L1, suggesting that L1 is the primary output location of L1 NDNF-INs (right) (19 slices, 3 animals).

(B) Optogenetic identification of the postsynaptic partners of L1 NDNF-INs in acute slices (top left). L1 and L2/3 INs were identified by nuclear mCherry expression (Peron et al., 2015) and L2/3 PNs by morphology. Calibration of ChR-2 expressing L1 NDNF-IN stimulation (bottom; top right shows an example trace). The chosen irradiance (gray lines, 45 mW/mm^2) elicited 1.2 action potentials per pulse (0.5 ms, n = 10).

(C) Average IPSCs in ChR-2 negative L1 INs (gray, n = 12), L2/3 PNs (black, n = 24), and L2/3 INs (red, n = 11).

(legend continued on next page)

previously generated bacterial artificial chromosome/clone (BAC)-transgenic line in which EGFP expression is driven from the *Ndnf* locus (Gong et al., 2003) and found that it faithfully recapitulates the endogenous expression of *Ndnf* in L1 (Figures S1I and S1J), whereas ectopic labeling was observed in deeper layers (Figure S1E). Finally, we addressed whether *Ndnf* is a useful marker for L1 INs outside of the auditory cortex. Analogous experiments in the pre- and infra-limbic areas of the prefrontal cortex (PL and IL, respectively) indicated that *Ndnf* expression is highly enriched in L1 INs (Figures S2A and S2B) and displays similar co-localization with other markers as in the auditory cortex (Figures S2C–S2E). Further experiments indicate that L1 NDNF-INs are faithfully addressed by both mouse alleles in the prefrontal cortex (Figure S2F). Together, these data demonstrate that our newly generated mouse lines allow for the specific labeling and manipulation of L1 NDNF-INs in the adult cortex.

L1 NDNF-INs Mediate Long-Lasting Inhibition of PN Dendrites

As a first step toward understanding how signaling by L1 NDNF-INs affects the function of the local circuit in the auditory cortex, we determined the localization of their output synapses. AAV-mediated transduction of Ndnf-Ires-CreERT2 mice with a synaptophysin-GFP fusion protein (Oh et al., 2014) revealed that by far the greatest output of these INs remains within L1 (Figures 2A and S3A). In addition, a second smaller and broader peak of synapse density is present approximately 300 μm from the pia spanning L3 and L4. To identify the postsynaptic partners of these synapses, we crossed Ndnf-Ires-CreERT2 animals with a mouse line labeling inhibitory INs (Peron et al., 2015). After AAV-mediated expression of channelrhodopsin-2 (ChR-2), this enabled reliable and precise light activation of L1 NDNF-INs in acute brain slices of adult auditory cortex (Figure 2B) that elicited inhibitory postsynaptic current (IPSCs) in ChR-2 negative L1 INs, L2/3 Ins, and L2/3 PNs (Figures 2C and 2D) with indistinguishable, short latencies (Figure S3D). While inputs to L1 INs and L2/3 PNs displayed similarly slow rise and decay times, the kinetics of IPSCs in L2/3 INs was significantly faster (Figures 2C and 2D). The strongest input was observed in L2/3 PNs, which together with the observed synapse localization indicates that one important effect of L1 NDNF-INs may be inhibition of distal PN dendrites within L1. Consistent with this, inputs to L2/3 PNs were also observed when optogenetic stimulation was restricted to L1 under block of action potential firing (Figures 2E and 2F; Petreanu et al., 2009), directly demonstrating distal dendritic inhibition. Together, these data indicate that L1 NDNF-IN output synapses are concentrated in L1 and display broad connectivity to other circuit elements and reveal inhibition of distal PN dendrites as a major consequence of L1 NDNF-IN activation.

A second, intensely studied source of inhibition in distal PN dendrites derives from the projection of SST-positive Martinotti cells to L1 (Figure 2G, Higley, 2014; Yavorska and Wehr, 2016). We therefore next asked how the two forms of dendritic inhibition might differ. Despite greater amplitudes of SST-IN inputs to L2/3 PNs (which might partly be due to more efficient optogenetic stimulation of these cells; Figure S3I), we found that L1 NDNF-IN IPSCs mediate greater charge transfer due to their much more prolonged kinetics (Figures 2H and 2I). In addition, L1 NDNF-IN input displayed slower rise times and onset latencies than SST IPSCs (Figures 2I and S3J), which were not due to differences in recording quality (Figure S3N). The long decay times of L1 NDNF-IN IPSCs may indicate a contribution of $GABA_B$ receptors at these synapses, which have been demonstrated to contribute strongly to transmission from NGCs (Tamás et al., 2003). Indeed, application of the selective $GABA_B$ receptor antagonist CGP 55845 (3 μM) markedly reduced the decay time of IPSCs mediated by L1 NDNF-INs, whereas SST input recorded under the same conditions remained unchanged (Figures 2J, 2K, and S3K). Importantly, inhibition from L1 NDNF-INs displayed much slower rise and decay times than SST IPSCs even under $GABA_B$ receptor block (Figures 2J and 2L), suggesting additional sources for these kinetic differences (see Discussion). Together, the present results indicate that inhibition from two distinct sources controls the activity of PN dendrites and identify L1 NDNF-INs as a genetically addressable source of inhibition in distal PN dendrites that differs markedly from the well-understood SST Martinotti cell input in terms of kinetics and underlying receptors. Consistent with the interpretation that the two forms of dendritic inhibition are optimized for low- versus high-frequency signaling, L1 NDNF-IN input to PNs also displayed stronger short-term depression than SST Martinotti cell input (Figure S3M). This is in line with the observed short-term depression of NGC synapses (Capogna and Pearce, 2011; Oláh et al., 2009; Overstreet-Wadiche and McBain, 2015; Tamás et al., 2003) and may suggest that in addition to the differences described here, inhibition from L1 NDNF-INs and SST-INs could also contribute to oscillations in different frequency bands.

(D) Comparison of L1 NDNF-IN-mediated IPSCs in the different postsynaptic populations. Note the greater amplitude and charge of IPSCs in L2/3 PNs and the faster rise and decay in L2/3 INs (Kruskal-Wallis H-test with Dunn's multiple comparison).

(E) Optogenetic activation of L1 NDNF-IN synapse selectively in L1 under action potential block (1 μM tetrodotoxin [TTX], 100 μM 4-AP).

(F) Input to L2/3 PNs is targeted to their distal dendrites located in L1.

(G) Comparison of NDNF and SST-IN input to the distal dendrites of L2/3 PNs. Note that the two datasets are from different experiments.

(H) Average IPSCs evoked by SST- (green, n = 13) and NDNF-IN stimulation (blue, n = 24).

(I) IPSCs mediated by L1 NDNF-INs showed greater charge transfer and longer rise and decay times compared to SST inhibition (Mann-Whitney test).

(J) IPSCs from SST- (green, n = 7) and L1 NDNF-INs (blue, n = 9) in baseline and after bath application of the selective $GABA_B$ receptor antagonist CGP 55845 (3 μM, brown, normalized).

(K) $GABA_B$ receptor block accelerated the decay time of IPSCs mediated by L1 NDNF-INs but left SST-IN inhibition unaffected (Mann-Whitney test).

(L) Kinetic differences between NDNF- and SST-IN IPSCs persist under $GABA_B$ receptor block, indicating additional sources (Mann-Whitney test).

Data in (B), (C), (F), (H), and (J) represent mean ± SEM; other plots show range, quartiles, and median. (D, I, K, and L) $^*p < 0.05$; $^{**}p < 0.01$; $^{***}p < 0.001$; $^{****}p < 0.0001$.

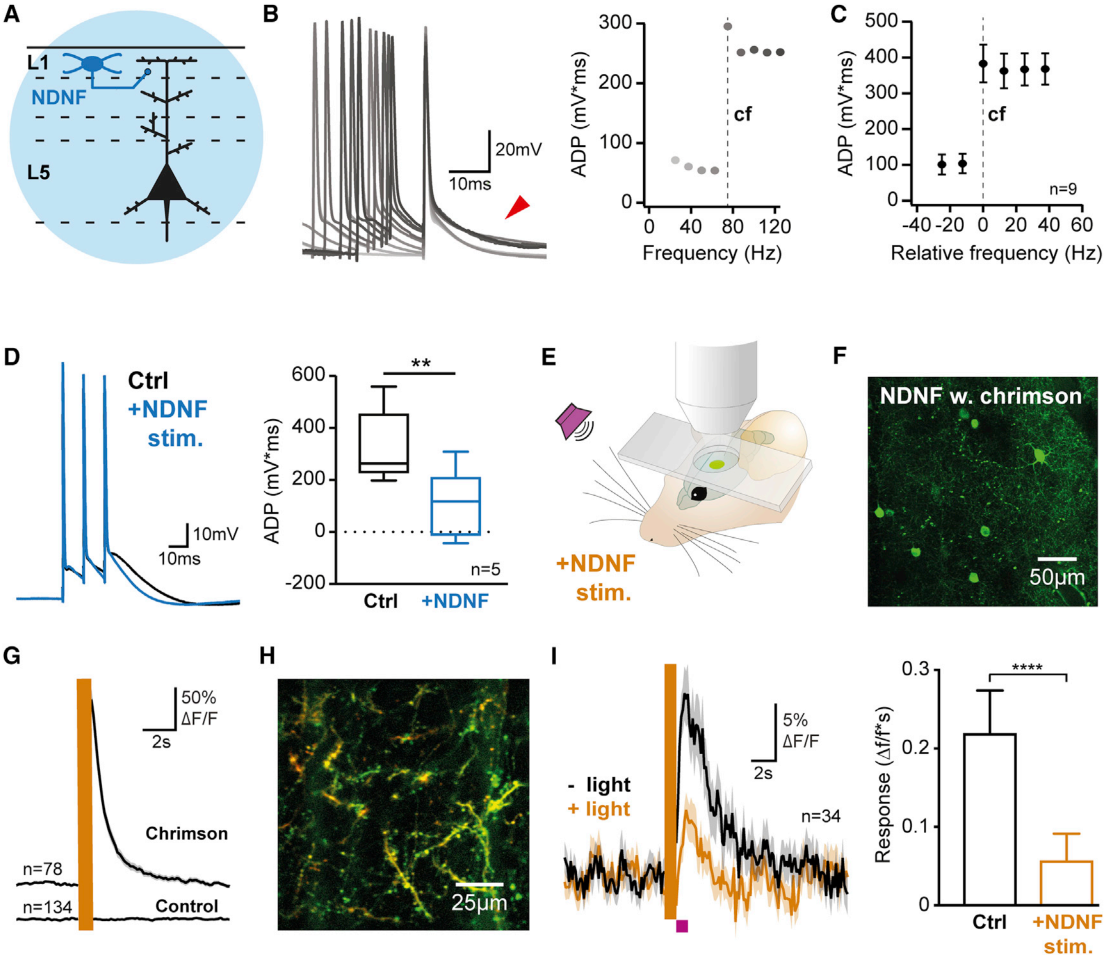

Figure 3. Layer 1 NDNF-Interneurons Control Activity in Pyramidal Neuron Dendrites

(A) Recordings from L5 PNs combined with optogenetic stimulation of L1 NDNF-INs.

(B) Stimulation of a L5 PN at increasing frequencies (3 action potentials, 25–125 Hz, light to dark gray) causes a sharp increase in the afterdepolarization (ADP, arrowhead, quantification on right) that constitutes the critical frequency of the neurons (dashed line) and correlates with dendritic spike initiation (Larkum et al., 1999a).

(C) Quantified data aligned to the critical frequency of each neuron (dashed line) reveals highly supralinear dependence of the ADP on action potential frequency (n = 9).

(D) Activation of L1 NDNF-INs (4 pulses at 40 Hz, ending 50–100 ms before last action potential, n = 5, paired t test) significantly reduced the ADP. This indicates that inhibition from L1 NDNF-INs powerfully controls the initiation of PN dendritic spikes in acute brain slices.

(E) *In vivo* 2-photon imaging in auditory cortex of awake mice combined with sensory stimulation (magenta, 5 white noise bursts, 100 ms duration, delivered at 5 Hz) and optogenetic activation of L1 NDNF-INs (yellow, 594 nm).

(F) Field of view during *in vivo* imaging of L1 NDNF-INs co-expressing GCaMP6s (green) and the optogenetic effector Chrimson in Ndnf-Ires-FlpO mice.

(G) Optogenetic activation (yellow) elicited strong responses in L1 NDNF-INs expressing Chrimson (top, n = 78) and no activity in animals that only expressed GCaMP6s (n = 134). These data demonstrate reliable optogenetic activation of L1 NDNF-INs in the awake auditory cortex.

(H) Field of view during *in vivo* imaging of distal PN dendrites in L1 expressing GCaMP6s (green) and tdTomato (red) used for motion correction. PNs were selectively labeled by a combination of retrograde Cre expression from subcortical regions (amygdala and striatum) and Cre-dependent expression of GCaMP6s and tdTomato in auditory cortex.

(I) Sensory responses (black) in dendritic branches that displayed significant activation by auditory stimulation (34 dendrites in 3 mice; see STAR Methods for details). Optogenetic activation of L1 NDNF-INs (yellow) immediately preceding auditory stimulation (magenta) caused a significant, long lasting reduction of dendritic responses (Wilcoxon test; see also Figures S4G–S4K). Together, these data demonstrate strong control of PN dendritic activity by L1 NDNF-INs *in vitro* and in awake animals.

Data in (D) represent range, quartiles, and median; other plots show mean ± SEM. (D and I) $^{**}p < 0.01$; $^{****}p < 0.0001$.

L1 NDNF-INs Control Activity in PN Dendrites

Our data suggest that in addition to PN disinhibition via L2/3 INs (Figures 2C, 2D, 5D, S3C, and S3D; Letzkus et al., 2011, 2015), a second important function of L1 INs may be to control the firing of dendritic spikes in distal PN dendrites, which are exquisitely sensitive to $GABA_B$ receptor activation (Larkum et al., 1999b; Palmer et al., 2012; Pérez-Garci et al., 2006). To directly address this, we elicited dendritic spikes in adult auditory cortex L5 PNs *in vitro* by action potential bursts of increasing frequency (Figures 3A and 3B). In line with previous results, the

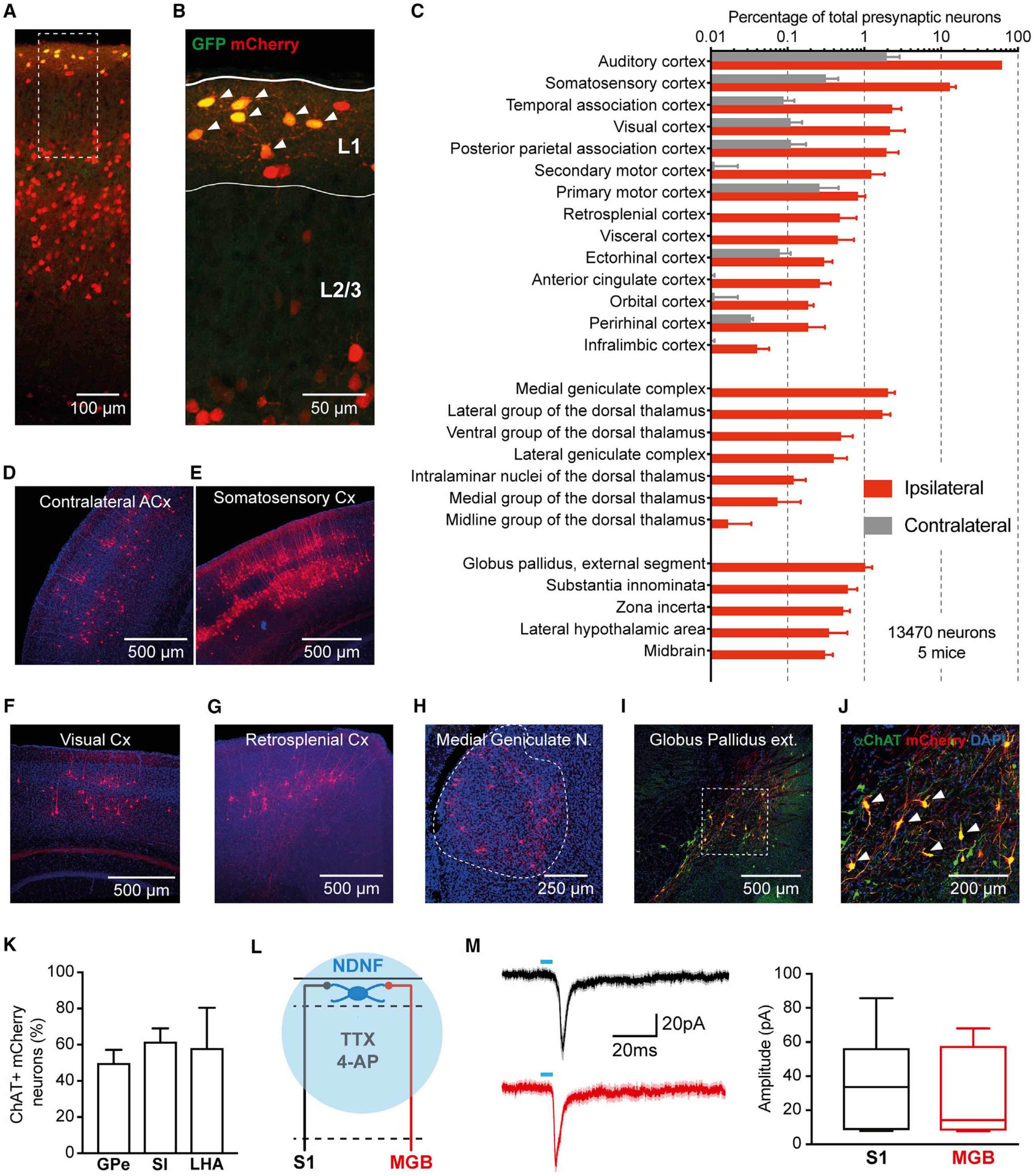

Figure 4. Brain-wide Sources of Synaptic Input to Auditory Cortex Layer 1 NDNF-Interneurons

(A) Representative image of the injection site in the adult auditory cortex. L1 NDNF-INs were made competent for rabies virus by injection of AAV-synP-DIO-sTpEpB (Kohara et al., 2014) and subsequent tamoxifen induction in Ndnf-Ires-CreERT2 mice. After 4–5 weeks of expression time, RV-dG-mCherry was injected at the same site. Note localization of starter cells expressing both GFP and mCherry in L1 and presynaptic partners in both L1 and deeper layers.

(B) Magnified view of the area indicated in (A). Starter L1 NDNF-INs are marked by arrowheads.

(legend continued on next page)

afterdepolarization (ADP) of the burst increased in a highly supralinear fashion with stimulation frequency (Figures 3B, 3C, and S4C), indicating the critical frequency beyond which a dendritic spike is elicited (Larkum et al., 1999a). Importantly, preceding optogenetic activation of L1 NDNF-INs strongly reduced the ADP at supracritical frequencies (Figures 3D and S4B) while leaving the ADP at subcritical frequencies unaffected (Figure S4B). These data demonstrate control of distal dendritic electrogenesis in L1 by NDNF-INs.

To determine whether L1 NDNF-INs also exert control over sensory responses of distal PN dendrites in L1 in the awake animal, we employed *in vivo* 2-photon imaging (Figure 3E). In order to obtain sparse labeling of PNs that is critical for dendritic calcium imaging, together with optogenetic control over NDNF-INs, we injected a retrograde vector carrying Cre recombinase into subcortical targets of auditory cortex in Ndnf-Ires-FlpO mice (amygdala and striatum). This allowed us to selectively express the calcium indicator GCaMP6s (Chen et al., 2013) along with tdTomato used for motion correction in PNs. At the same time, a Flp-dependent AAV was injected in auditory cortex to enable expression of the optogenetic activator Chrimson in NDNF-INs, which is ideal for combination with 2-photon imaging due to its sensitivity and red-shifted excitation spectrum (Klapoetke et al., 2014). We first validated this approach by expressing both GCaMP6s and Chrimson in NDNF-INs (Figure 3F). Stimulation triggered large calcium transients in Chrimson-expressing NDNF-INs and no detectable responses in NDNF-INs expressing only GCaMP6s (Figures 3G and S4F), indicating successful optogenetic stimulation. We next imaged the activity of PN dendrites in L1 (Figure 3H; n = 130 dendrites in 3 mice). Sensory stimulation (5 white noise bursts, 100 ms duration, presented at 5 Hz) elicited significant responses in 34 out of 130 dendritic segments (Figure 3I; see STAR Methods for details). Importantly, preceding optogenetic activation of L1 NDNF-INs strongly reduced these dendritic responses (Figure 3I), and similar results were obtained when taking all recorded dendrites into consideration (Figure S4I; n = 130). While this experiment alone cannot rule out a contribution of somatic inhibition to the observed reduction in dendritic activity, when taken together with our observation that L1 NDNF-IN output synapses are strongly enriched in L1, where they directly contact PN distal dendrites (Figures 2A, 2E, and 2F), these data provide strong evidence for direct control over dendritic activity by L1 NDNF-INs in the intact animal. This inhibition lasted several seconds (Figure S4H), consistent with the prolonged time course of L1 NDNF-IN inhibition we found in our slice recordings (Figures 2H and 2I). Interestingly, larger responses were more strongly suppressed by NDNF-IN input (Figure S4J), which together with the data from L5 PNs suggests that inhibition from NDNF-INs controls the firing of spikes in distal PN dendrites in L1.

Brain-wide Sources of Synaptic Input to Auditory Cortex L1 NDNF-INs

Having established the local circuit elements and subcellular compartments targeted by L1 NDNF-INs in the auditory cortex, we next aimed to obtain a precise understanding of the brain-wide synaptic inputs to these cells. We therefore employed monosynaptically restricted tracing with modified rabies viral vectors (Wickersham et al., 2007). Consistent with our validation (Figures S1E and S1H), starter cells targeted by AAV injection in Ndnf-Ires-CreERT2 were highly enriched in auditory cortex L1 (Figures 4A, 4B, S5A, and S5B). Monosynaptically connected neurons (n = 13,470, 5 mice) were found locally in the auditory cortex and in a number of additional brain areas both ipsi- and contralaterally (Figures 4C–4H and S5). In line with the known afferent organization of L1, we observed a range of cortical feedback projections from sensory areas (somatosensory, visual), motor (primary and secondary) association areas (e.g., retrosplenial, temporal association), and frontal areas (anterior cingulate, infralimbic). In addition, several thalamic nuclei provide input to auditory cortex L1 NDNF-INs, including the medial geniculate nucleus and the dorsal thalamus. Finally, several additional brain regions were labeled, most notably areas that contain cholinergic neurons such as the globus pallidus externus and substantia innominate. Given that only a fraction of neurons in these areas are cholinergic (Gritti et al., 2006), we performed an antibody staining for choline acetyl transferase (ChAT) and found that approximately half of the presynaptic neurons are cholinergic (Figures 4I–4K). Finally, to ensure that rabies vector labeled neurons are indeed monosynaptically connected, we performed an anterograde, physiological validation on the strongest cortical input from outside of auditory cortex (somatosensory cortex) and the strongest thalamic afferent connection (medial geniculate body). *In vitro* recordings from L1 NDNF-INs showed monosynaptic input in both cases (n = 7 each; Figure 4M). We note that while this approach demonstrates functional monosynaptic connectivity, the amplitude of the

(C) Brain-wide input map to auditory cortex L1 NDNF-INs obtained by referencing mCherry cells (13,470 neurons from 5 animals) to the Allen Brain Atlas (Fürth et al., 2018). This analysis reveals a large number of cortical (top), thalamic (center), and other areas (bottom) that provide afferent input to auditory cortex L1 NDNF-INs.

(D–H) Representative images of the indicated areas.

(I and J) Image of mCherry-expressing neurons in globus pallidus externus counterstained for ChAT, identifying several input neurons in this area to be cholinergic (I); arrowheads in (J), high magnification.

(K) Approximately half of the mCherry-expressing neurons in the globus pallidus externus (GPe), substantia innominate (SI), and lateral hypothalamic area (LHA) were ChAT positive, revealing substantial cholinergic input to auditory cortex L1 NDNF-INs from these areas.

(L) Anterograde physiological validation of the strongest cortical (somatosensory cortex [S1]) and strongest thalamic (medial geniculate body [MGB]) input sources.

(M) Optogenetic stimulation of these axons under action potential block by TTX and 4-AP to prevent polysynaptic input elicits excitatory postsynaptic current (EPSCs) of comparable amplitude in auditory cortex L1 NDNF-INs (n = 7 each, $p > 0.05$, unpaired t test), confirming that rabies virus tracing identifies true synaptic connectivity.

Data in (M) represent range, quartiles, and median; other plots show mean ± SEM.

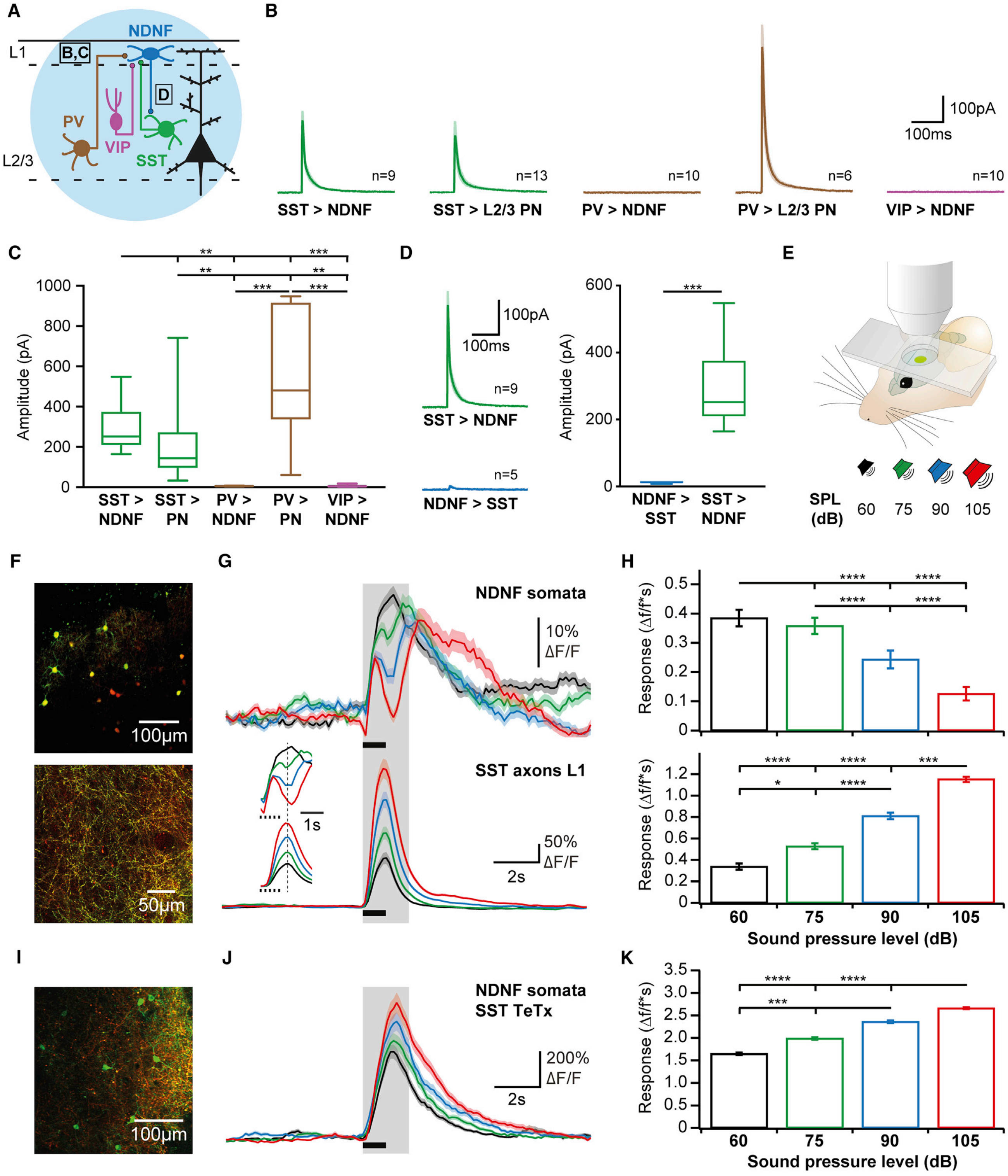

Figure 5. Inhibitory Control of Layer 1 NDNF-Interneuron Activity in the Auditory Cortex

(A) Optogenetic identification of inhibitory inputs to L1 NDNF-INs. ChR-2 was expressed in SST-, PV-, or VIP-INs, and whole-cell recordings were performed in acute slices from genetically identified L1 NDNF-INs (Gong et al., 2003) and neighboring L2/3 PNs for comparison.

(B) Average IPSCs in L1 NDNF-INs (n = 9 from SST, n = 10 from PV, and n = 10 from VIP) and L2/3 PNs (n = 13 from SST and n = 6 from PV).

(legend continued on next page)

postsynaptic currents depends on several experimental factors (including ChR-2 expression levels and what fraction of presynaptic axons remain viable after slicing) and is therefore likely not indicative of the true connection strength in the intact brain (Petreanu et al., 2009). Together, these data significantly extend previous observations on long-range inputs to unidentified L1 INs (Bennett et al., 2012; Cruikshank et al., 2012; Letzkus et al., 2011; Palmer et al., 2012; Poorthuis et al., 2018; Zhu and Zhu, 2004). In particular, compared to similar experiments on PV-, SST-, and VIP-INs in somatosensory cortex (Wall et al., 2016), our results indicate that L1 NDNF-INs receive input from a larger range of brain areas encoding contextual, top-down information. In turn, this suggests that the activity of L1 NDNF-INs may strongly be governed by internally generated signals such as those occurring during memory expression.

Inhibitory Control of L1 NDNF-IN Activity

In addition to excitation, inhibitory input from other INs can dominantly shape the activity and function of different IN types in cortical circuits (Kepecs and Fishell, 2014; Letzkus et al., 2015; Lovett-Barron and Losonczy, 2014; Overstreet-Wadiche and McBain, 2015; Wester and McBain, 2014). To determine the local inhibitory inputs to L1 NDNF-INs, we crossed Cre lines for SST, VIP, and PV (Hippenmeyer et al., 2005; Taniguchi et al., 2011) to mice expressing EGFP under the *Ndnf* promoter (Figures 5A, S1E, S1I, and S1J; Gong et al., 2003). Cre-dependent expression of ChR-2 allowed reliable activation of these different IN types in auditory cortex acute slices (Figures S3I and S6C), and recordings were performed from L1 NDNF-INs and from neighboring L2/3 PNs for comparison. As expected, activation of both SST- and PV-INs elicited large IPSCs in the PNs (Figures 5B and 5C), with faster rise times and onset latencies for PV input, as predicted from its perisomatic localization (Figure S6E). In contrast, analogous recordings from L1 NDNF-INs revealed no measurable inhibition from either PV- or VIP-positive populations but strong input from SST-INs (Figures 5B and 5C). The strength of this input was equal to that measured in neighboring L2/3 PNs in terms of amplitude and charge transfer. These results indicate that out of the populations tested, SST-INs are the only source of inhibition in L1 NDNF-INs, consistent with the projection of SST-positive Martinotti cells to L1 (Higley, 2014; Yavorska and Wehr, 2016) and previous results in unidentified L1 INs (Pfeffer et al., 2013). To test whether this interaction is reciprocal, we employed a cross of SST-Ires-Cre and Ndnf-Ires-FlpO mice. TdTomato was expressed in SST-INs to target the recordings, whereas the optogenetic activator Chrimson was expressed in L1 NDNF-INs (Figures 3F and 3G). Optogenetic stimulation of L1 NDNF-INs elicited IPSCs in SST-INs (Figure 5D), indicating bidirectional communication between the two IN types (Jiang et al., 2015). However, inhibition from NDNF- to SST-INs was significantly weaker than in the opposite direction (Figure 5D). To address whether this result may be due to stimulation efficiency, we recorded L1 NDNF-IN input in neighboring L2/3 PNs in each one of these experiments. The amplitude of IPSCs evoked after Flp-dependent expression of Chrimson was indistinguishable from Cre-mediated ChR-2 expression (Figure S6F), indicating similar stimulation efficiency. Together, these data indicate largely unidirectional information flow from SST-INs to L1 NDNF-INs. Intriguingly, this connectivity motif indicates that the two distinct forms of dendritic inhibition derived from NDNF- and SST-INs identified above function in parallel and interact at the level of L1 NDNF-INs.

To address whether inhibition from SST Martinotti cells is a dominant factor controlling the sensory responses of L1 NDNF-INs in the intact circuit, we next performed *in vivo* 2-photon calcium imaging in auditory cortex of awake mice. Motivated by results from visual cortex indicating that visual stimuli of increasing size recruit progressively stronger responses of SST-INs (Adesnik et al., 2012), we performed an analogous

(C) L1 NDNF-INs receive strong inhibition from SST-INs similar to L2/3 PNs but no input from PV- or VIP-INs (Kruskal-Wallis H-test with Dunn's multiple comparison).

(D) The opposite connection direction was addressed in a cross of SST-Ires-Cre and Ndnf-Ires-FlpO animals, allowing expression of Chrimson for light stimulation in L1 NDNF-INs in combination with tdTomato expression in SST-INs to target these cells for whole-cell recordings. Light stimulation elicited IPSCs in neighboring PNs with amplitudes indistinguishable from those evoked with ChR-2 (Figure S6F), indicating efficient recruitment of L1 NDNF-INs. In contrast, input from L1 NDNF-INs to SST-INs (n = 5) displayed much smaller amplitudes than in the opposite direction (right, n = 9, unpaired t test), indicating that inhibition is largely unidirectional from SST- to NDNF-INs.

(E) *In vivo* 2-photon imaging in auditory cortex of awake mice during presentation of white noise (5 bursts, 100 ms duration, delivered at 5 Hz) at different sound pressure levels.

(F) Fields of view during *in vivo* imaging of L1 NDNF-INs (top) and axons derived from SST-INs (bottom) in the auditory cortex L1. L1 NDNF-INs expressed GCaMP6s due to the better signal-to-noise ratio, whereas SST axons were imaged using either GCaMP6s or GCaMP6f for better temporal resolution. Both populations also expressed tdTomato for motion correction.

(G) Average responses of L1 NDNF-IN somata (top, 95 neurons in 5 mice) and SST axons (bottom, 11 regions in 11 mice, 8 with GCaMP6f, and 2 with GCaMP6s) during auditory stimulation at different sound pressure levels (indicated by the black bar; color code in E). Inset: responses in the quantified time window (2 s after stimulus onset) at higher temporal resolution. Note that the excitatory peak in SST axons (bottom, dashed line) coincides with the local minimum in L1 NDNF-INs (top). Importantly, similar data were obtained with somatic imaging of SST-INs (Figures S6H–S6J).

(H) Quantification of the response integral during 2 s after stimulation onset (gray shading in G for L1 NDNF-INs [top] and SST axons [bottom]). While SST axon responses increased with increasing stimulus intensity, L1 NDNF-INs displayed the opposite relationship (>6 trials per intensity, Friedman test with Dunn's multiple comparison).

(I) To test whether input from SST-INs causes the observed inhibition of L1 NDNF-INs at higher stimulus intensities, we crossed SST-Ires-Cre and Ndnf-Ires-FlpO animals, allowing expression of GCaMP6s in L1 NDNF-INs in combination with expression of tetanus toxin light chain (TeTx) and tdTomato in SST-INs.

(J) Silencing of synaptic release from SST-INs converted the responses of L1 NDNF-INs (n = 38 neurons in 4 mice) from decreasing with stimulus intensity in controls (G, top) to increasing.

(K) Quantification of the response integral during 2 s after stimulation onset (gray shading in J; Friedman test with Dunn's multiple comparison).

Data in (C) and (D) represent range, quartiles, and median; other plots show mean ± SEM. (C, D, H, and K) *p < 0.05; **p < 0.01; ***p < 0.001; ****p < 0.0001.

experiment by presenting trains of auditory stimuli of increasing sound pressure levels to the animals in head fixation (Figure 5E). To target selectively the output of Martinotti cells, which reside in both supra- and infragranular layers (Yavorska and Wehr, 2016), as opposed to other types of SST-INs, we imaged the axons of SST-INs in L1 using GCaMP6 (as done by Lovett-Barron et al., 2014). These data show that stimuli of increasing intensity recruit progressively stronger responses of SST-IN axons in L1 (Figures 5F–5H and S6K), consistent with the interpretation that, like in visual cortex, this form of inhibition is proportional to the activity of the local PN network (Adesnik et al., 2012). Importantly, analogous experiments in SST-IN somata in L2/3 produced very similar results (Figures S6H–S6K), indicating the validity of the axonal imaging approach. Moreover, presynaptic calcium influx is tightly coupled to neurotransmitter release (Bucurenciu et al., 2008), suggesting that the axonal calcium responses are directly related to inhibitory transmission from SST-INs in L1. Strikingly, the same experiment on L1 NDNF-INs produced the opposite result, with the greatest activation by the lowest-intensity stimulus and successively smaller responses for louder sounds during the 2 s after stimulation onset (Figures 5F–5H and S6K). While stimulation at all sound pressure levels caused initial excitation of L1 NDNF-INs, louder stimuli elicited successively larger inhibition that coincided with the peak of SST axon activation (Figure 5G). In contrast, L1 NDNF-IN responses after this time window, when SST-IN activity has largely decayed, did not depend on sound pressure level. Together with the robust inhibition observed in slice recordings, these data indicate that the activity of L1 NDNF-INs may be under tight control by SST Martinotti cells also in the awake auditory cortex. To causally test this hypothesis, we combined Flp-mediated expression of GCaMP6 in L1 NDNF-INs with Cre-dependent expression of tetanus toxin in SST-INs to silence selectively the output of these cells. In contrast to control experiments (Figures 5F–5H), L1 NDNF-INs showed no discernible inhibition during presentation of high intensity stimuli in these experiments (Figure 5J). Moreover, L1 NDNF-INs displayed sensory responses that increased with stimulus intensity (Figure 5K), in stark contrast to the decrease observed when SST inhibition was intact (Figure 5H). Together, these data therefore demonstrate that input from SST-INs is a dominant factor shaping the activity of L1 NDNF-INs in the intact circuit. Functionally, this circuit organization indicates that SST inhibition can effectively override the impact of L1 NDNF-INs, successively replacing NDNF input to PN dendrites by SST input at greater stimulus intensities. Furthermore, the long onset latencies of SST inhibition (Figure S6L) are consistent with the recruitment of SST-INs by local recurrent excitation (Adesnik et al., 2012; Yavorska and Wehr, 2016). In contrast, the initial excitation of L1 NDNF-INs was significantly faster (Figure S6L), suggesting that it derives from a different source, such as long-range inputs from thalamus or the cholinergic basal forebrain (Figure 4). These results thus raise the possibility that in addition to the different postsynaptic effects of L1 NDNF-INs and SST-INs (Figure 2) and the inhibitory interaction shown here, these populations may also differ in the excitatory afferents that recruit them and could therefore also serve different behavioral functions.

Learning-Related Plasticity of Sensory Responses in L1 NDNF-INs

To directly address the contribution of inhibition from L1 NDNF-INs and SST-INs to a defined behavioral function, we subjected mice to associative auditory fear conditioning, a paradigm for which a large body of work has reported plasticity of stimulus responses in the auditory cortex (LeDoux, 2000; Weinberger, 2007). While different forms of fear memory acquisition and expression have furthermore been found to depend on processing in the auditory cortex, the strongest evidence to date has been obtained for discriminative fear conditioning with frequency-modulated sweeps as conditioned stimuli (CS), in which conditioned freezing and conditioned licking suppression depend on auditory cortex activity (Gillet et al., 2018; Letzkus et al., 2011). We therefore combined this behavior with *in vivo* 2-photon calcium imaging (Figure 6A). This approach allowed us to record the responses of the same L1 NDNF-INs during habituation, when the CS are neutral, and again during retrieval of the fear memory. Similar to previous work (Letzkus et al., 2011), fear memory measured as freezing in a second retrieval session under freely behaving conditions was strong and discriminative, indicating successful memory acquisition (Figures 6B and S7B–S7D). To establish a readout of fear expression in head fixation, we determined the change in pupil diameter in response to presentation of CS during habituation and retrieval. These data indicate greater pupil responses after fear learning (Figures 6C and S7E) and a positive correlation between freezing and the difference in pupil response between retrieval and habituation (Figure 6D), indicating that under these conditions, pupil diameter can be used as a proxy for successful fear memory retrieval in head fixation.

During habituation, the population of L1 NDNF-INs recorded in these animals (133 neurons in 8 mice) responded to the CSs with similar response integrals and amplitudes (Figures 6E–6H and S7F), with ~30% of cells strongly activated (CS− 39/133 neurons, CS+ 37/133 neurons) by the stimuli and a smaller fraction of significantly inhibited neurons (CS− 13/133 neurons, CS+ 17/133 neuron; Figures S7H–S7K; STAR Methods). Imaging the same cells again after fear conditioning revealed a pronounced increase in CS+ responses during fear memory expression and a smaller potentiation also for the CS−, which elicits intermediate fear levels (Figures 6F–6H, S7F, and S7G). This potentiation of CS responses was mediated by both stronger excitatory responses (Figures S7H and S7I) in a greater number of L1 NDNF-INs (CS− 61/133 neurons, CS+ 57/133 neurons strongly excited) and a reduction of inhibitory transients (Figures S7J and S7K; CS− and CS+ 5/133 neurons strongly inhibited). These data demonstrate that recall of an aversive memory is associated with a pronounced increase in L1 NDNF-IN responses. To further address whether this effect is indeed related to memory expression, we performed analogous experiments on animals that underwent pseudoconditioning (CSs and foot shocks presented unpaired, n = 5). These mice displayed no CS-evoked freezing behavior (Figure 6I), and in contrast to the above observations, the CS responses of L1 NDNF-INs were strongly *reduced* during the second imaging session (Figures 6J and S7G), indicating that repeated CS presentation without fear learning decreases L1 NDNF-IN signaling, potentially by

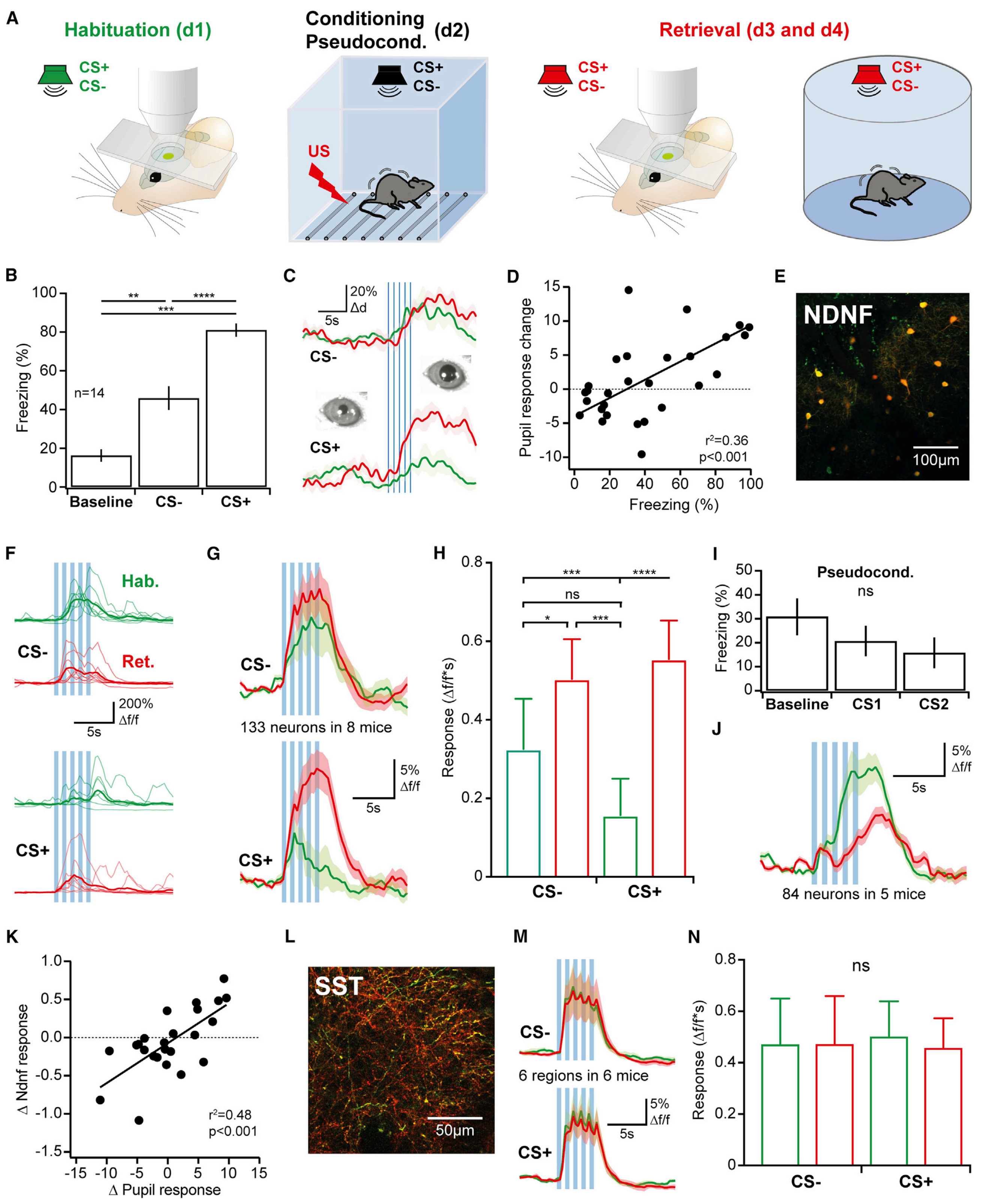

(legend on next page)

nonassociative habituation. Interestingly, animals that underwent fear conditioning but failed to form a stable memory (n = 2, criterion: <40% freezing for the CS+; Figure S7D) also showed a trend for decreased L1 NDNF-IN responses (Figure S7L), further underpinning the interpretation that potentiation of L1 NDNF-IN responses is related to fear memory. Strikingly, when we subsequently combined the data from fear and pseudoconditioned mice (n = 12), we found that the change in L1 NDNF-IN responses to a CS correlates with the CS's learned relevance as measured by either pupil responses or freezing (Figures 6K and S7M). Together, these data demonstrate that encoding of sensory information by L1 NDNF-INs is robustly and dynamically modulated by the animal's experience and that along with stronger disinhibition through L2/3 INs, fear memory expression may be associated with increased inhibition of PN dendrites within L1. However, the level of dendritic inhibition also depends on SST-INs. We therefore performed analogous experiments imaging the CS responses of SST axons in L1 (see validation in Figures S6H–S6K; Lovett-Barron et al., 2014). SST axons showed highly reliable CS responses during habituation that were completely unaffected by fear memory expression (Figures 6L–6N). While this result does not preclude plasticity within this population of axons, it does indicate that net inhibition from this source remains stable. This suggests that potentiation of L1 NDNF-IN-mediated inhibition occurs against a stable backdrop of inhibition from Martinotti cells and reveals that inhibition from these two sources plays distinct roles also during behavioral expression of memory.

DISCUSSION

Using a selective genetic marker for L1 INs in conjunction with two novel mouse lines, viral tracing, slice recordings, optogenetics, *in vivo* 2-photon calcium imaging, and associative fear memory, our results identify L1 NDNF-INs as a strong and highly plastic source of inhibition of distal PN dendrites that differs from and is complementary to SST Martinotti cells at several levels of organization. One distinguishing feature is the slow time course and strong $GABA_B$ receptor contribution of inhibition from L1 NDNF-INs, which is consistent with previous work on morphologically identified NGCs in L1 (Chu et al., 2003; Jiang et al., 2013, 2015; Palmer et al., 2012; Wozny and Williams, 2011) and other circuits (Tamás et al., 2003; Capogna and Pearce, 2011; Overstreet-Wadiche and McBain, 2015) and with co-expression of the NGC markers *Reln*, *Npy*, and *nNos* in L1 NDNF-INs. In line with this, recent slice recordings have suggested that L1 NDNF-INs in the mouse and human neocortex correspond to neurogliaform cells (Poorthuis et al., 2018; Tasic et al., 2016), together indicating that *Ndnf* will enable future investigations into these so-far little-understood cells and may also facilitate translation of these insights to the human brain. In addition, our results place L1 NDNF-INs into the inhibitory wiring diagram of the neocortex. While our main focus was a functional comparison of these cells to SST-INs as the second genetically addressable source of inhibition in distal PN dendrites, L1 NDNF-INs and unidentified L1 INs also share certain attributes with the better-understood VIP-INs; both IN types have been implicated in disinhibition (Figure 2; Letzkus et al., 2011, 2015; Pi et al., 2013) are recruited by cholinergic input (Kepecs and Fishell, 2014; Letzkus et al., 2015; Poorthuis et al., 2018) as well as a range of top-down afferents (Figure 4; Wall et al., 2016) and encode contextual signals such as reinforcement (Letzkus et al., 2011; Pi et al., 2013). A more precise understanding of the similarities and differences between NDNF- and VIP-INs, in particular in defined behavioral paradigms, should therefore be obtained in future work. In addition, more work is

Figure 6. Plasticity of Layer 1 NDNF-Interneuron Responses after Associative Learning

(A) Discriminative auditory fear conditioning in combination with awake *in vivo* 2-photon imaging. Trains of frequency-modulated sweeps of opposite modulation direction (counterbalanced between experiments) were used as conditioned stimuli (CS).

(B) Freezing behavior of the fear-conditioned animals presented in (E)–(N) in a freely behaving memory retrieval session on day 3 or 4 indicates strong, discriminative fear memory (CS+: 8 animals up sweeps, 6 animals down sweeps, one-way ANOVA $F(1.7, 21.7) = 56.1$, $p < 0.0001$; Tukey's multiple comparison test).

(C) Example pupil diameter response to CS presentation during habituation (green) and memory retrieval (red, sweep onset blue lines) in head fixation.

(D) The change in pupil response for the CSs (response integral retrieval minus integral habituation) correlated with freezing to the stimuli (both fear and pseudoconditioned mice shown), demonstrating that pupil responses can be used as a fear readout under the microscope.

(E) Field of view for *in vivo* imaging of NDNF-INs in the auditory cortex L1 of awake, head-fixed mice (conditional expression of GCaMP6s [green] and tdTomato [red] in Ndnf-Ires-CreERT2).

(F) Responses of an example L1 NDNF-IN before and after fear conditioning (thin traces represent single trials and thick traces averages).

(G) Average CS responses of all imaged L1 NDNF-INs (133 neurons in 8 mice, CS+: 58 neurons up sweeps, 75 neurons down sweeps) showing a modest increase for the CS− and strong potentiation of CS+ responses.

(H) Quantification of response integral. Both CS− and CS+ responses were significantly potentiated after fear conditioning, whereas no difference was observed during habituation ($p = 0.93$, Friedman test with Dunn's multiple comparison).

(I) Freezing behavior of pseudoconditioned animals (n = 5). Note absence of CS evoked freezing (one-way ANOVA $F(1.5, 5.8) = 3.2$, $p > 0.05$).

(J) Average CS responses of all imaged L1 NDNF-IN in mice from (I) (84 neurons in 5 mice, CS1 and CS2 combined) showing a decrease in responses for these stimuli.

(K) Correlation between the response change in L1 NDNF-INs due to fear conditioning (response integral retrieval minus integral habituation) and the change in pupil response elicited by that stimulus for fear and pseudoconditioned animals indicates that potentiation of L1 NDNF-IN correlates with learned stimulus relevance.

(L) Field of view during imaging of axons derived from SST-INs in the auditory cortex L1 (GCaMP6s, green; tdTomato, red).

(M) Average CS responses of all imaged SST axons (6 regions in 6 mice, CS+: 4 animal up sweeps, 2 animals down sweeps) showing no change with fear conditioning.

(N) Quantification of response integral ($p > 0.05$, Friedman test with Dunn's multiple comparison).

Data are shown as mean ± SEM. (B and H) *$p < 0.05$; **$p < 0.01$; ***$p < 0.001$; ****$p < 0.0001$.

required to determine whether L1 NDNF-INs in other areas, such as the prefrontal cortex, show similarities to the present results on connectivity and learning-related plasticity. Importantly, the slow form of GABAergic volume transmission mediated by L1 NDNF-INs may not only target PN dendrites but also control the release probability of local synapses via presynaptic $GABA_B$ receptors (Capogna and Pearce, 2011; Chittajallu et al., 2013; Oláh et al., 2009; Overstreet-Wadiche and McBain, 2015; Price et al., 2008; Tamás et al., 2003). Thus, an additional potentially important function of these cells may be to control how afferent information is received in L1.

Previous work has implicated L1 INs in brief breaks in the perisomatic excitation-inhibition balance contributing to memory acquisition (Jiang et al., 2013, 2015; Letzkus et al., 2011), and evidence from the barrel cortex indicates that inhibition from NGCs can also serve to selectively constrain perisomatic feed-forward inhibition (Chittajallu et al., 2013). While disinhibition has recently emerged as a conserved circuit motif for learning and memory (Hattori et al., 2017; Letzkus et al., 2015), our results add to this view by showing that disinhibition via L2/3 INs is accompanied by a concomitant increase in dendritic inhibition. This result adds to important work demonstrating that dynamic reallocation of inhibition along the subcellular compartments of PNs is a key mechanism that controls firing patterns, oscillations, and the impact of different excitatory afferent pathways in cortical PNs (Somogyi et al., 2013). In contrast to proximal inhibition, dendritic inhibition can leave the somatic membrane potential unaffected due to strong electrotonic attenuation and the underlying channels (Palmer et al., 2012) and can even increase the robustness of somatic stimulus encoding (Egger et al., 2015). The emerging working hypothesis that needs to be tested in future research is therefore that L1 NDNF-IN input may enable strong and reliable somatic stimulus representation while at the same time powerfully controlling dendritic spikes. Firing of dendritic spikes in turn has been linked to induction of synaptic plasticity in distal PN dendrites (Cichon and Gan, 2015; Golding et al., 2002; Kampa et al., 2007; Letzkus et al., 2006). Given the adaptive value of protecting in particular fear memories from change or degradation over time by additional plasticity induction, L1 NDNF-INs may therefore serve to constrain dendritic plasticity induction after memory formation is complete.

Our results identify inhibition from SST Martinotti cells controlled by recurrent excitation as a dominant factor that shapes L1 NDNF-IN activity. Together with our observation that auditory cortex L1 NDNF-INs receive long-range, top-down information implicated in memory from a larger number of brain areas compared to similar data from SST-INs in the somatosensory cortex (Wall et al., 2016), this gives rise to the testable hypothesis that L1 NDNF-INs are able to integrate and compare top-down information from these long-range sources with the ongoing processing of bottom-up input in the local circuit encoded by SST-INs, extending the available evidence for the proposed function of L1 INs in predictive coding (Bastos et al., 2012). In particular, likely sources for the observed learning-related potentiation of L1 NDNF-IN sensory responses include afferents from the cholinergic basal forebrain (Letzkus et al., 2011, 2015; Pi et al., 2013; Poorthuis et al., 2018) as well as thalamic (LeDoux, 2000; Weinberger, 2007) and cortico-cortical input (Makino and Komiyama, 2015). On the other hand, the inhibitory connection motif predicts that under conditions of reduced SST activity, such as engagement in operant behavior (Kato et al., 2015; Makino and Komiyama, 2015), L1 NDNF-INs can contribute relatively more to dendritic inhibition, similar to our observations during memory retrieval. Conversely, when stimuli become less salient during habituation, SST-IN responses are increased (Kato et al., 2015), leading to inhibition of L1 NDNF-INs that may contribute to the observed reduction of stimulus encoding after pseudoconditioning. Together, this suggests that SST inhibition dominates in conditions of weak and imprecise stimulus encoding in PNs, whereas recruitment of L1 NDNF-INs occurs when sensory input is currently relevant to the animal.

STAR★METHODS

Detailed methods are provided in the online version of this paper and include the following:

- KEY RESOURCES TABLE
- CONTACT FOR REAGENT AND RESOURCE SHARING
- EXPERIMENTAL MODEL AND SUBJECT DETAILS
  - ○ Animals and generation of Ndnf-IRES-CreERT2 and Ndnf-IRES-FlpO mice
- METHOD DETAILS
  - ○ Fluorescent InSitu Hybridization (FISH)
  - ○ Perfusions, immunohistochemistry and morphological analysis of fluorescent reporter expression
  - ○ Surgery
  - ○ Rabies tracing
  - ○ Virus injection and implantation of cranial windows
  - ○ Fear conditioning
- QUANTIFICATION AND STATISTICAL ANALYSIS
- DATA AND SOFTWARE AVAILABILITY
  - ○ AudioGame
  - ○ EyeTracker and Camera Acquisition
  - ○ Processing of calcium imaging data
  - ○ FreezingScoring

SUPPLEMENTAL INFORMATION

Supplemental Information includes seven figures and can be found with this article online at https://doi.org/10.1016/j.neuron.2018.09.001.

A video abstract is available at https://doi.org/10.1016/j.neuron.2018.09.001#mmc3.

ACKNOWLEDGMENTS

We thank all members of the Letzkus and Spiegel labs, E. Schuman, M.S. Fustinana Gueler, J. Gjorgjieva, and members of the FENS Kavli Network of Excellence for comments and discussions; A. Wrana for outstanding technical assistance; S. Junek and F. Vollrath for help with image processing; F. Kretschmer and G. Tushev for assistance with programming and data analysis; L.L. Looger, J. Akerboom, D.S. Kim, and the GENIE Project at Janelia Farm for making GCaMP available; K. Deisseroth, E.S. Boyden, and H. Zeng for generously sharing reagents; and M.E. Greenberg for his generous support in the early stages of this project. This work was supported by the Max Planck Society, the European Research Council (StG 335587 to J.J.L.), the Minna James Heineman Foundation (to I.S. and J.J.L.), the Human Frontier Science

Program (to M.B.P.), the Boehringer Ingelheim Fonds (to L.E.), and DFG SPP1665 (to K.K.C.). I.S. is the incumbent of the Friends and Linda and Richard Price Career Development Chair and a scholar in the Zuckerman STEM leadership program.

AUTHOR CONTRIBUTIONS

J.J.L. and I.S. initiated and conceived the project. Experiments and data analyses were performed by D.A., D.K., and I.S. with help from K.M., R.B.P., and J.J.L. (Figures 1, S1, and S2); R.B.P., K.M., M.B.P., and J.J.L. (Figures 2, 3A–3D, 4K–4L, 5A–5D, S3, S4A–S4C, and S6A–S6F); E.A., M.B.P., L.E., and J.J.L. (Figures 3E–3I, 5F–5K, 6, S4D–S4K, S6G–S6L, and S7); D.-L.P., R.B.P., and J.J.L., with rabies vector provided by K.-K.C. and M.F.E. (Figures 4A–4J and S5). J.J.L. wrote the manuscript with input from all authors. All authors contributed to experimental design and interpretation and commented on the manuscript.

DECLARATION OF INTERESTS

The authors declare no competing interests.

Received: May 10, 2018
Revised: August 9, 2018
Accepted: August 31, 2018
Published: September 27, 2018

REFERENCES

Adesnik, H., Bruns, W., Taniguchi, H., Huang, Z.J., and Scanziani, M. (2012). A neural circuit for spatial summation in visual cortex. Nature *490*, 226–231.

Bastos, A.M., Usrey, W.M., Adams, R.A., Mangun, G.R., Fries, P., and Friston, K.J. (2012). Canonical microcircuits for predictive coding. Neuron *76*, 695–711.

Bennett, C., Arroyo, S., Berns, D., and Hestrin, S. (2012). Mechanisms generating dual-component nicotinic EPSCs in cortical interneurons. J. Neurosci. *32*, 17287–17296.

Bucurenciu, I., Kulik, A., Schwaller, B., Frotscher, M., and Jonas, P. (2008). Nanodomain coupling between Ca2+ channels and Ca2+ sensors promotes fast and efficient transmitter release at a cortical GABAergic synapse. Neuron *57*, 536–545.

Cadwell, C.R., Palasantza, A., Jiang, X., Berens, P., Deng, Q., Yilmaz, M., Reimer, J., Shen, S., Bethge, M., Tolias, K.F., et al. (2016). Electrophysiological, transcriptomic and morphologic profiling of single neurons using Patch-seq. Nat. Biotechnol. *34*, 199–203.

Capogna, M., and Pearce, R.A. (2011). GABA A,slow: causes and consequences. Trends Neurosci. *34*, 101–112.

Cauller, L. (1995). Layer I of primary sensory neocortex: where top-down converges upon bottom-up. Behav. Brain Res. *71*, 163–170.

Chen, T.W., Wardill, T.J., Sun, Y., Pulver, S.R., Renninger, S.L., Baohan, A., Schreiter, E.R., Kerr, R.A., Orger, M.B., Jayaraman, V., et al. (2013). Ultrasensitive fluorescent proteins for imaging neuronal activity. Nature *499*, 295–300.

Chittajallu, R., Pelkey, K.A., and McBain, C.J. (2013). Neurogliaform cells dynamically regulate somatosensory integration via synapse-specific modulation. Nat. Neurosci. *16*, 13–15.

Chu, Z., Galarreta, M., and Hestrin, S. (2003). Synaptic interactions of late-spiking neocortical neurons in layer 1. J. Neurosci. *23*, 96–102.

Cichon, J., and Gan, W.B. (2015). Branch-specific dendritic Ca(2+) spikes cause persistent synaptic plasticity. Nature *520*, 180–185.

Cruikshank, S.J., Ahmed, O.J., Stevens, T.R., Patrick, S.L., Gonzalez, A.N., Elmaleh, M., and Connors, B.W. (2012). Thalamic control of layer 1 circuits in prefrontal cortex. J. Neurosci. *32*, 17813–17823.

Douglas, R.J., and Martin, K.A. (2004). Neuronal circuits of the neocortex. Annu. Rev. Neurosci. *27*, 419–451.

Egger, R., Schmitt, A.C., Wallace, D.J., Sakmann, B., Oberlaender, M., and Kerr, J.N. (2015). Robustness of sensory-evoked excitation is increased by inhibitory inputs to distal apical tuft dendrites. Proc. Natl. Acad. Sci. USA *112*, 14072–14077.

Felleman, D.J., and Van Essen, D.C. (1991). Distributed hierarchical processing in the primate cerebral cortex. Cereb. Cortex *1*, 1–47.

Fürth, D., Vaissière, T., Tzortzi, O., Xuan, Y., Märtin, A., Lazaridis, I., Spigolon, G., Fisone, G., Tomer, R., Deisseroth, K., et al. (2018). An interactive framework for whole-brain maps at cellular resolution. Nat. Neurosci. *21*, 139–149.

Gillet, S.N., Kato, H.K., Justen, M.A., Lai, M., and Isaacson, J.S. (2018). Fear learning regulates cortical sensory representations by suppressing habituation. Front. Neural Circuits *11*, 112.

Golding, N.L., Staff, N.P., and Spruston, N. (2002). Dendritic spikes as a mechanism for cooperative long-term potentiation. Nature *418*, 326–331.

Gong, S., Zheng, C., Doughty, M.L., Losos, K., Didkovsky, N., Schambra, U.B., Nowak, N.J., Joyner, A., Leblanc, G., Hatten, M.E., and Heintz, N. (2003). A gene expression atlas of the central nervous system based on bacterial artificial chromosomes. Nature *425*, 917–925.

Gorski, J.A., Talley, T., Qiu, M., Puelles, L., Rubenstein, J.L., and Jones, K.R. (2002). Cortical excitatory neurons and glia, but not GABAergic neurons, are produced in the Emx1-expressing lineage. J. Neurosci. *22*, 6309–6314.

Gritti, I., Henny, P., Galloni, F., Mainville, L., Mariotti, M., and Jones, B.E. (2006). Stereological estimates of the basal forebrain cell population in the rat, including neurons containing choline acetyltransferase, glutamic acid decarboxylase or phosphate-activated glutaminase and colocalizing vesicular glutamate transporters. Neuroscience *143*, 1051–1064.

Habib, N., Avraham-Davidi, I., Basu, A., Burks, T., Shekhar, K., Hofree, M., Choudhury, S.R., Aguet, F., Gelfand, E., Ardlie, K., et al. (2017). Massively parallel single-nucleus RNA-seq with DroNc-seq. Nat. Methods *14*, 955–958.

Hattori, R., Kuchibhotla, K.V., Froemke, R.C., and Komiyama, T. (2017). Functions and dysfunctions of neocortical inhibitory neuron subtypes. Nat. Neurosci. *20*, 1199–1208.

Helmchen, F., Svoboda, K., Denk, W., and Tank, D.W. (1999). In vivo dendritic calcium dynamics in deep-layer cortical pyramidal neurons. Nat. Neurosci. *2*, 989–996.

Higley, M.J. (2014). Localized GABAergic inhibition of dendritic Ca(2+) signalling. Nat. Rev. Neurosci. *15*, 567–572.

Hippenmeyer, S., Vrieseling, E., Sigrist, M., Portmann, T., Laengle, C., Ladle, D.R., and Arber, S. (2005). A developmental switch in the response of DRG neurons to ETS transcription factor signaling. PLoS Biol. *3*, e159.

Jiang, X., Wang, G., Lee, A.J., Stornetta, R.L., and Zhu, J.J. (2013). The organization of two new cortical interneuronal circuits. Nat. Neurosci. *16*, 210–218.

Jiang, X., Shen, S., Cadwell, C.R., Berens, P., Sinz, F., Ecker, A.S., Patel, S., and Tolias, A.S. (2015). Principles of connectivity among morphologically defined cell types in adult neocortex. Science *350*, aac9462.

Kampa, B.M., Letzkus, J.J., and Stuart, G.J. (2007). Dendritic mechanisms controlling spike-timing-dependent synaptic plasticity. Trends Neurosci. *30*, 456–463.

Kato, H.K., Gillet, S.N., and Isaacson, J.S. (2015). Flexible sensory representations in auditory cortex driven by behavioral relevance. Neuron *88*, 1027–1039.

Kepecs, A., and Fishell, G. (2014). Interneuron cell types are fit to function. Nature *505*, 318–326.

Klapoetke, N.C., Murata, Y., Kim, S.S., Pulver, S.R., Birdsey-Benson, A., Cho, Y.K., Morimoto, T.K., Chuong, A.S., Carpenter, E.J., Tian, Z., et al. (2014). Independent optical excitation of distinct neural populations. Nat. Methods *11*, 338–346.

Kohara, K., Pignatelli, M., Rivest, A.J., Jung, H.Y., Kitamura, T., Suh, J., Frank, D., Kajikawa, K., Mise, N., Obata, Y., et al. (2014). Cell type-specific genetic and optogenetic tools reveal hippocampal CA2 circuits. Nat. Neurosci. *17*, 269–279.

Kuang, X.L., Zhao, X.M., Xu, H.F., Shi, Y.Y., Deng, J.B., and Sun, G.T. (2010). Spatio-temporal expression of a novel neuron-derived neurotrophic factor (NDNF) in mouse brains during development. BMC Neurosci. *11*, 137.

Larkum, M.E. (2013). The yin and yang of cortical layer 1. Nat. Neurosci. *16*, 114–115.

Larkum, M.E., Kaiser, K.M., and Sakmann, B. (1999a). Calcium electrogenesis in distal apical dendrites of layer 5 pyramidal cells at a critical frequency of back-propagating action potentials. Proc. Natl. Acad. Sci. USA *96*, 14600–14604.

Larkum, M.E., Zhu, J.J., and Sakmann, B. (1999b). A new cellular mechanism for coupling inputs arriving at different cortical layers. Nature *398*, 338–341.

LeDoux, J.E. (2000). Emotion circuits in the brain. Annu. Rev. Neurosci. *23*, 155–184.

Letzkus, J.J., Kampa, B.M., and Stuart, G.J. (2006). Learning rules for spike timing-dependent plasticity depend on dendritic synapse location. J. Neurosci. *26*, 10420–10429.

Letzkus, J.J., Wolff, S.B., Meyer, E.M., Tovote, P., Courtin, J., Herry, C., and Lüthi, A. (2011). A disinhibitory microcircuit for associative fear learning in the auditory cortex. Nature *480*, 331–335.

Letzkus, J.J., Wolff, S.B., and Lüthi, A. (2015). Disinhibition, a circuit mechanism for associative learning and memory. Neuron *88*, 264–276.

Lovett-Barron, M., and Losonczy, A. (2014). Behavioral consequences of GABAergic neuronal diversity. Curr. Opin. Neurobiol. *26*, 27–33.

Lovett-Barron, M., Kaifosh, P., Kheirbek, M.A., Danielson, N., Zaremba, J.D., Reardon, T.R., Turi, G.F., Hen, R., Zemelman, B.V., and Losonczy, A. (2014). Dendritic inhibition in the hippocampus supports fear learning. Science *343*, 857–863.

Madisen, L., Zwingman, T.A., Sunkin, S.M., Oh, S.W., Zariwala, H.A., Gu, H., Ng, L.L., Palmiter, R.D., Hawrylycz, M.J., Jones, A.R., et al. (2010). A robust and high-throughput Cre reporting and characterization system for the whole mouse brain. Nat. Neurosci. *13*, 133–140.

Major, G., Larkum, M.E., and Schiller, J. (2013). Active properties of neocortical pyramidal neuron dendrites. Annu. Rev. Neurosci. *36*, 1–24.

Makino, H., and Komiyama, T. (2015). Learning enhances the relative impact of top-down processing in the visual cortex. Nat. Neurosci. *18*, 1116–1122.

Mardinly, A.R., Spiegel, I., Patrizi, A., Centofante, E., Bazinet, J.E., Tzeng, C.P., Mandel-Brehm, C., Harmin, D.A., Adesnik, H., Fagiolini, M., and Greenberg, M.E. (2016). Sensory experience regulates cortical inhibition by inducing IGF1 in VIP neurons. Nature *531*, 371–375.

Oh, S.W., Harris, J.A., Ng, L., Winslow, B., Cain, N., Mihalas, S., Wang, Q., Lau, C., Kuan, L., Henry, A.M., et al. (2014). A mesoscale connectome of the mouse brain. Nature *508*, 207–214.

Oláh, S., Füle, M., Komlósi, G., Varga, C., Báldi, R., Barzó, P., and Tamás, G. (2009). Regulation of cortical microcircuits by unitary GABA-mediated volume transmission. Nature *461*, 1278–1281.

Overstreet-Wadiche, L., and McBain, C.J. (2015). Neurogliaform cells in cortical circuits. Nat. Rev. Neurosci. *16*, 458–468.

Palmer, L.M., Schulz, J.M., Murphy, S.C., Ledergerber, D., Murayama, M., and Larkum, M.E. (2012). The cellular basis of GABA(B)-mediated interhemispheric inhibition. Science *335*, 989–993.

Pérez-Garci, E., Gassmann, M., Bettler, B., and Larkum, M.E. (2006). The GABAB1b isoform mediates long-lasting inhibition of dendritic Ca2+ spikes in layer 5 somatosensory pyramidal neurons. Neuron *50*, 603–616.

Peron, S.P., Freeman, J., Iyer, V., Guo, C., and Svoboda, K. (2015). A cellular resolution map of barrel cortex activity during tactile behavior. Neuron *86*, 783–799.

Petreanu, L., Mao, T., Sternson, S.M., and Svoboda, K. (2009). The subcellular organization of neocortical excitatory connections. Nature *457*, 1142–1145.

Pfeffer, C.K., Xue, M., He, M., Huang, Z.J., and Scanziani, M. (2013). Inhibition of inhibition in visual cortex: the logic of connections between molecularly distinct interneurons. Nat. Neurosci. *16*, 1068–1076.

Pi, H.J., Hangya, B., Kvitsiani, D., Sanders, J.I., Huang, Z.J., and Kepecs, A. (2013). Cortical interneurons that specialize in disinhibitory control. Nature *503*, 521–524.

Poorthuis, R.B., Muhammad, K., Wang, M., Verhoog, M.B., Junek, S., Wrana, A., Mansvelder, H.D., and Letzkus, J.J. (2018). Rapid neuromodulation of layer 1 interneurons in human neocortex. Cell Rep. *23*, 951–958.

Price, C.J., Scott, R., Rusakov, D.A., and Capogna, M. (2008). GABA(B) receptor modulation of feedforward inhibition through hippocampal neurogliaform cells. J. Neurosci. *28*, 6974–6982.

Sanz, E., Yang, L., Su, T., Morris, D.R., McKnight, G.S., and Amieux, P.S. (2009). Cell-type-specific isolation of ribosome-associated mRNA from complex tissues. Proc. Natl. Acad. Sci. USA *106*, 13939–13944.

Somogyi, P., Katona, L., Klausberger, T., Lasztóczi, B., and Viney, T.J. (2013). Temporal redistribution of inhibition over neuronal subcellular domains underlies state-dependent rhythmic change of excitability in the hippocampus. Philos. Trans. R. Soc. Lond. B Biol. Sci. *369*, 20120518.

Stuart, G.J., and Spruston, N. (2015). Dendritic integration: 60 years of progress. Nat. Neurosci. *18*, 1713–1721.

Takahashi, N., Oertner, T.G., Hegemann, P., and Larkum, M.E. (2016). Active cortical dendrites modulate perception. Science *354*, 1587–1590.

Tamás, G., Lorincz, A., Simon, A., and Szabadics, J. (2003). Identified sources and targets of slow inhibition in the neocortex. Science *299*, 1902–1905.

Taniguchi, H., He, M., Wu, P., Kim, S., Paik, R., Sugino, K., Kvitsiani, D., Fu, Y., Lu, J., Lin, Y., et al. (2011). A resource of Cre driver lines for genetic targeting of GABAergic neurons in cerebral cortex. Neuron *71*, 995–1013.

Tasic, B., Menon, V., Nguyen, T.N., Kim, T.K., Jarsky, T., Yao, Z., Levi, B., Gray, L.T., Sorensen, S.A., Dolbeare, T., et al. (2016). Adult mouse cortical cell taxonomy revealed by single cell transcriptomics. Nat. Neurosci. *19*, 335–346.

Wall, N.R., De La Parra, M., Sorokin, J.M., Taniguchi, H., Huang, Z.J., and Callaway, E.M. (2016). Brain-Wide Maps of Synaptic Input to Cortical Interneurons. J. Neurosci. *36*, 4000–4009.

Weinberger, N.M. (2007). Associative representational plasticity in the auditory cortex: a synthesis of two disciplines. Learn. Mem. *14*, 1–16.

Wester, J.C., and McBain, C.J. (2014). Behavioral state-dependent modulation of distinct interneuron subtypes and consequences for circuit function. Curr. Opin. Neurobiol. *29*, 118–125.

Wickersham, I.R., Lyon, D.C., Barnard, R.J., Mori, T., Finke, S., Conzelmann, K.K., Young, J.A., and Callaway, E.M. (2007). Monosynaptic restriction of transsynaptic tracing from single, genetically targeted neurons. Neuron *53*, 639–647.

Wozny, C., and Williams, S.R. (2011). Specificity of synaptic connectivity between layer 1 inhibitory interneurons and layer 2/3 pyramidal neurons in the rat neocortex. Cereb. Cortex *21*, 1818–1826.

Xu, N.L., Harnett, M.T., Williams, S.R., Huber, D., O'Connor, D.H., Svoboda, K., and Magee, J.C. (2012). Nonlinear dendritic integration of sensory and motor input during an active sensing task. Nature *492*, 247–251.

Yavorska, I., and Wehr, M. (2016). Somatostatin-expressing inhibitory interneurons in cortical circuits. Front. Neural Circuits *10*, 76.

Zeisel, A., Muñoz-Manchado, A.B., Codeluppi, S., Lönnerberg, P., La Manno, G., Juréus, A., Marques, S., Munguba, H., He, L., Betsholtz, C., et al. (2015). Brain structure. Cell types in the mouse cortex and hippocampus revealed by single-cell RNA-seq. Science *347*, 1138–1142.

Zhu, Y., and Zhu, J.J. (2004). Rapid arrival and integration of ascending sensory information in layer 1 nonpyramidal neurons and tuft dendrites of layer 5 pyramidal neurons of the neocortex. J. Neurosci. *24*, 1272–1279.

STAR★METHODS

KEY RESOURCES TABLE

REAGENT or RESOURCE	SOURCE	IDENTIFIER
Antibodies		
Mouse-anti-NeuN (diluted 1:25)	MilliPore	Cat# MAB377; RRID: AB_2298772
Rabbit-anti-GFP, 488 conjugated (diluted 1:500)	LifeTechnologies	Cat# A21311; RRID: AB_221477
Rabbit-anti-RFP (diluted 1:500)	MBL Life science	Cat# PM005; RRID: AB_591279
Goat Anti-Mouse IgG (H+L) Alexa Fluor 647 (Highly Cross-Adsorbed) (diluted 1:250)	Life Technologies	Cat# A32728; RRID: AB_2633277
Goat Anti-Rabbit IgG (H+L) Alexa Fluor 647 (Highly Cross-Adsorbed) (diluted 1:1000)	Life Technologies	Cat# A-21245; RRID: AB_2535813
Goat-anti-ChAT (diluted 1:360)	MilliPore	Cat# AB144P-200UL; RRID: AB_90661
Donkey Anti-Goat IgG (H+L) Alexa Fluor 488 (diluted 1:1000)	Abcam	Cat# AB150129; RRID: AB_2687506
Bacterial and Virus Strains		
AAV2/1-CAG.Flex.GCaMP6s.WPRE.SV40	PennVector Core	Cat# AV-1-PV2818
AAV2/1.CAG.Flex.tdTomato.WPRE.bGH	PennVector Core	Cat# AV-1-ALL864
AAV2/1.CAG.Flex.GCaMP6f.WPRE.SV40	PennVector Core	Cat# AV-1-PV2816
AAV2/5.EF1a.DIO.hChR2(H134R)-EYFP.WPRE.hGH	PennVector Core	Cat# AV-5-20298P
AAV2/5.EF1a.dflox.hChR2(H134R)-mCherry.WPRE.hGH	PennVector Core	Cat# AV-5-20297P
AAV2/1-phSyn1-FLEX-tdTomato-T2A-SypEGFP-WPRE	Salk Vector Core	Cat# 51509
CAV2-CMV-Cre-SV40	Plateforme de Vectorolgie de Montpellier	CAV Cre
AAV2/1-EF1a-fDIO-ChrimsonR-wpre-sv40	Vector Biolabs	N/A
AAV2/1-EF1a-fDIO-GCamP6s-wpre-sv40	Vector Biolabs	N/A
AAV-DJ CMV DIO eGFP-2A-TeNT	Stanford Medicine Vector Core	Cat# GWC-AAV-71
AAV2/5-synP-DIO-sTpEpB	UNC Vector Core	Cat# AV6118CD
RV-SAD dG-mcherry	Klaus Conzelmann	N/A
AAV2/1-EF1a-fDIO-EYFP-WPRE	UNC Vector Core	Cat# AV6154B
Chemicals, Peptides, and Recombinant Proteins		
Manual Assay RNAscope Mm-Ndnf-C1	ACD Bio	Cat# 447471
Manual Assay RNAscope Mm-Npas1-C1	ACD Bio	Cat# 468851
Manual Assay RNAscope Mm-Ndnf-C2	ACD Bio	Cat# 447471-C2
Manual Assay RNAscope Mm-Pvalb-C2	ACD Bio	Cat# 421931-C2
Manual Assay RNAscope Mm-Sst-C2	ACD Bio	Cat# 404631-C2
Manual Assay RNAscope Mm-Vip-C2	ACD Bio	Cat# 415961-C2
Manual Assay RNAscope Mm-Reln-C2	ACD Bio	Cat# 405981-C2
Manual Assay RNAscope Mm-Npy-C2	ACD Bio	Cat# 313321-C2
Manual Assay RNAscope Mm-Calb2-C2	ACD Bio	Cat# 313641-C2
Manual Assay RNAscope Mm-Nos1-C2	ACD Bio	Cat# 437651-C2
Manual Assay RNAscope Mm-Slc17a7-C2	ACD Bio	Cat# 416631-C2
Manual Assay RNAscope tdTomato-C2	ACD Bio	Cat# 317041-C2
Manual Assay RNAscope Egfp-C3	ACD Bio	Cat# 400281-C3
Manual Assay RNAscope Mm-Gad1-C3	ACD Bio	Cat# 400951-C3
Critical Commercial Assays		
RNAscope Multiplex Fluorescent Reagent Kit	ACD Bio	Cat# 320850

(*Continued on next page*)

Continued		
REAGENT or RESOURCE	SOURCE	IDENTIFIER
Experimental Models: Organisms/Strains		
Mouse: C57BL/6J		N/A
Mouse: *Ndnf*$^{Cre\text{-}ERT2}$	Ivo Spiegel	N/A
Mouse: *Ndnf*^{FlpO}	Johannes Letzkus and Ivo Spiegel	N/A
Mouse: *Sst*$^{tm2.1(cre)Zjh}$/J	The Jackson Laboratory	Cat# 013044; RRID: IMSR_JAX:013044
Mouse: *Vip*$^{tm1(cre)Zjh}$/J	The Jackson Laboratory	Cat# 010908; RRID: IMSR_JAX:010908
Mouse: B6;129P2-*Pvalb*$^{tm1(cre)Arbr}$/J	The Jackson Laboratory	Cat# 008069; RRID: IMSR_JAX:008069
Mouse: B6.Cg-*Gt(ROSA)26Sor*$^{tm9(CAG\text{-}tdTomato)Hze}$/J	The Jackson Laboratory	Cat# 007909; IMSR_JAX:007909
Mouse: B6;129S-*Gad2*$^{tm1.1Ksvo}$/J	The Jackson Laboratory	Cat# 023140; IMSR_JAX:023140
Mouse: Mouse: *Ndnf*^{Egfp} (= B6;FVB-Tg(A930038C07Rik-EGFP) HV74Gsat/Mmucd, backcrossed to C57Bl6)	MMRRC	Cat# 030028-UCD; RRID: MMRRC_030028-UCD
B6(Cg)-*Calb2*$^{tm1(cre)Zjh}$/J	The Jackson Laboratory	Cat# 010774; RRID: IMSR_JAX:010774
Software and Algorithms		
MATLAB	MathWorks	N/A
Prism	GraphPad	N/A
Fiji	https://imagej.net/Welcome	N/A
Imaris	BitPlane	N/A
pClamp	Molecular Devices	N/A

CONTACT FOR REAGENT AND RESOURCE SHARING

Further information and requests for resources and reagent should be directed to and will be fulfilled by the Lead Contact, Johannes J. Letzkus (Johannes.Letzkus@brain.mpg.de).

EXPERIMENTAL MODEL AND SUBJECT DETAILS

Animals and generation of Ndnf-IRES-CreERT2 and Ndnf-IRES-FlpO mice

Male C57BL6/J mice, and mouse lines maintained in C57Bl6/J background (1.5-6 months old) were housed under a 12 h light/dark cycle, and provided with food and water *ad libitum*. After surgical procedures, mice were individually housed. All animal procedures were executed in accordance with institutional guidelines, and approved by the prescribed authorities (Regierungspräsidium Darmstadt and Institutional Animal Care and Use Committee [IACUC] at the Weizmann Institute of Science).

The Ndnf-IRES-CreERT2 and Ndnf-IRES-FlpO alleles were generated at Cyagen Biosciences Inc using standard techniques and protocols for homologous recombination in ES-cells. The targeting vector was cloned from two BAC-clones (RP23-274L3, RP23-84F6) and ES-cells were a proprietary cell-line on a C57Bl6-background. Proper targeting of the *Ndnf*-locus was validated by Southern Blotting and by PCR on genomic DNA that was extracted from ES-cells. Validated correctly targeted ES-cells were then used to generate chimeric founder mice that were subsequently crossed to a deleter strain (on a C57Bl6 background) to remove a Neomycin-resistance cassette (flanked by FLP-sites) that was contained in the original targeting construct. The Ndnf-IRES-CreERT2 and Ndnf-IRES-FlpO colonies were then established from the resulting heterozygous offspring that did not contain the deleter construct. The lines were maintained by backcrosses to C57BL6/J and by PCR-based genotyping.

METHOD DETAILS

Fluorescent InSitu Hybridization (FISH)

FISH was done with the RNAscope system (Advanced Cell Diagnostic) essentially as described (Mardinly et al., 2016); all probes and reagents were from Advanced Cell Diagnostic. Brains of 8-week old wild-type C57Bl6 mice were dissected, fresh frozen on dry ice in Tissue-Tek OCT compound (Fisher Scientific) and stored at -80°C until use. 8 μm thick coronal cryosections of the frozen brains were prepared on a cryostat (Leica) and mounted onto Superfrost Plus object slides. FISH itself was done according to the manufacturer's instructions and the hybridized slides were imaged and analyzed as described below.

For analyzing the distribution of IN subtypes across auditory cortex and prefrontal cortex (pre- and infralimbic areas, PL and IL, i.e., distance from pia of *Ndnf*-, *Pv*-, *Sst*-, *Vip*-expressing neurons), the hybridized sections were imaged on an Olympus VS120 Virtual

Slide Microscope with a 20x objective (auditory cortex) or an Zeiss LSM800 confocal microscope with a 63x objective (prefrontal cortex). Image settings were kept constant throughout a given experiment for each channel/marker/probe and multiple fields-of-view were stitched into one compound image. The compound images of each brain section were then imported into Imaris, where the area of the auditory cortex was marked; in this area, the distance of each marker-expressing cell from the pia was measured.

For analyzing the co-expression of Ndnf with highly-expressed IN-subtype markers in the auditory cortex (Figures 1F–1H), brain sections were imaged on an Olympus VS120 Virtual Slide Microscope with a 20x objective and multiple fields-of-view were stitched into one compound image (imaging settings were kept constant throughout a given experiment for each channel/marker/probe). The compound images were then imported to Imaris, where the auditory cortex and its layers were labeled. *Ndnf* and other marker-expressing cells were labeled using the "Spots" module which marks a spot on each identified cell. Ndnf-spots were then co-localized with the respective marker spots, and all the spots were counted. For analyzing the co-expression of Ndnf with weakly-expressed genes (i.e., *Reln*, *Npy*, *Calb2*, *Npas1*), the auditory or prefrontal cortices in each section were imaged on a Zeiss LSM800 confocal microscope with a 63x objective and multiple fields-of-view were "stitched" into one compound image (imaging settings were kept constant throughout a given experiment for each channel/marker/probe). Compound images of each auditory cortex were then imported to Photoshop, and additional layers were created for each probe (i.e., one layer for *Ndnf* and one layer for the subtype marker in each compound image). The cells positive for each probe were then marked with a dot in the respective new layer and the layers containing the dots were compiled into a separate image file together with the DAPI-layer. These dots were then counted manually. For each combination of probes (*Ndnf* together with each of the subtype markers), two auditory cortices from four animals were analyzed (a total of 6-8 auditory cortices for each combination). For the combination of *Ndnf* together with *Npas1*, 14 cortices from 7 animals were analyzed due to the relatively low number of *Npas1*-expressing cells in L1.

Perfusions, immunohistochemistry and morphological analysis of fluorescent reporter expression

Mice were anesthetized with 10% ketamine and 1% xylazine in PBS and transcardially perfused with ice cold PBS for five minutes followed by fifteen minutes of cold 4% PFA in PBS. Brains were then dissected, post-fixed for 1 to 24 h at 4°C in 4% PFA, washed three times (10 minutes each) in cold PBS, and in a subset of experiments cryoprotected overnight in 20% sucrose in PBS at 4°C. The brains were then either frozen in Tissue-Tek Cryo-OCT compound (Fisher Scientific) on dry ice and stored at −80°C, or stored at 4°C in PBS. Coronal sections (10-15 μm thick) of auditory and prefrontal cortices in frozen tissue were cut using a Leica CM1950 cryostat, free-floating coronal sections (50-100 μm thick) were prepared with a Campden vibratome (5100mz) and used for subsequent experiments.

Immunostaining was done by blocking the slides for 1 hour in blocking buffer (PBS with 5% normal goat serum and 0.1% Triton X-100), staining the samples overnight in primary antibodies (diluted in blocking buffer, followed by three washes in PBS) and staining with secondary antibodies and Hoechst counterstain for 45 min at room-temperature or 24h at 4°C. The slides were then mounted in FluoromountG (Southern Biotech) and imaged on a Zeiss microscope (Axio Imager, LSM 710 or LSM 800). Antibodies are listed in STAR Methods. The following images are compounds obtained by 'stitching' of different fields-of-view: Figures 1C, 1E, 1I, 2A, 4A, 4B, 4D–4J, S1E–S1G, S2A, S3A, and S5A.

Surgery

Mice were anesthetized with isoflurane (induction: 4%, maintenance: 2%) in oxygen-enriched air (Oxymat 3, Weinmann, Hamburg, Germany) and fixed in a stereotaxic frame (Kopf Instruments, Tujunga, USA). Core body temperature was maintained at 37.5°C by a feed-back controlled heating pad (FHC, Bowdoinham, ME, USA). Analgesia was provided by local injection of ropivacain under the scalp (Naropin, AstraZeneca, Switzerland) and systemic injection of metamizol (100 mg/kg, i.p., Novalgin, Sanofi) and meloxicam (2 mg/kg, i.p., Metacam, Boehringer-Ingelheim, Ingelheim, Germany). Adeno-associated viral vectors (AAV, serotype 2/1 or 2/5, 100-500 nl) were injected from glass pipettes (tip diameter 10-20 μm) connected to a pressure ejection system (PDES-02DE-LA-2, NPI, Germany) into auditory cortex at the following coordinates: 2.46 mm posterior of bregma, 4.5 mm lateral of midline, depth below cortical surface varied for experiments. For MGB coordinates were 3.16 mm posterior bregma, 1.8 mm lateral of midline and 3.2 mm below cortical surface. Expression in Ndnf-Ires-CreERT2 animals was induced by intraperitoneal injections of tamoxifen (100-150 μl, 10 mg/ml, dissolved in 90% cornoil and 10% ethanol) on 4 consecutive days. We note that multiple tamoxifen doses are required for full induction. Experiments were performed after 1-3 months of expression time.

Rabies tracing

Ndnf-Ires-CreERT2 animals were injected with 100 nL AAV-synP-DIO-sTpEpB expressing TVA, eGFP and RV SAD B19 G protein in a Cre-dependent way in ACx as described above (see Surgery). Expression was induced by i.p. injection with tamoxifen the following 4 days. 4-5 weeks after this, 300 nL of EnvA-pseudotyped RV-dG-mCherry was injected into ACx during a second surgery. Importantly, RV injection without prior injection of AAV-synP-DIO-sTpEpB caused negligible labeling (1 neuron in 2 mice), indicating the specificity of the RV approach. One week later mice were transcardially perfused as described above. 60 μm coronal section were cut using a Leica vibratome (VT1000S), rinsed in PBS, incubated with blocking solution (3% bovine serum albumin and 0.2% Triton X-100 in PBS) for 2 h and afterward incubated with Goat-anti-ChAT (1:360) for 72 h at 4°C. Subsequently, sections were washed with PBS three times (10 min each) and incubated for 2 h at room temperature with fluorescent Donkey Anti-Goat IgG (H+L) Alexa Fluor 488 (1:1000). Finally, immuno-labeled sections were rinsed three times with PBS, mounted, covered with glass

coverslips, and imaged using a confocal microscope (LSM880, Carl Zeiss AG) with a 10x objective and 0.6-fold digital zoom. To quantify the cell number per animal, every third section of the entire brain was scanned using Zen software (Zeiss, Germany) and cells were counted using a custom written MATLAB software. To define the cell numbers in different brain regions images were registered to the Allen Brain Atlas (Fürth et al., 2018).

Virus injection and implantation of cranial windows

For implantation of cranial windows, a craniotomy was performed over the right auditory cortex using a sterile biopsy punch (3 mm, Integra Miltex). AAV2/1-CAG-flex-tdTomato-WPRE-bGH and AAV2/1-CAG-flex-GCaMP6s-WPRE-SV40 (1:1; Penn vector core) were co-injected at several sites in the craniotomy (300-500 nL total). For the sound intensity experiments in Figures 5F–5H and S6, SST-Cre mice were crossed to a floxed tdTomato strain (Ai9, Madisen et al., 2010) and injected with AAV2/1-CAG-flex-GCaMP6f-WPRE-SV40 or AAV2/1-CAG-flex-GCaMP6s-WPRE-SV40. For the experiment in Figures 3I–3K SST-Ires-Cre mice were crossed to NDNF-Ires-FlpO mice and injected with AAV2/1-CAG-flex-tdTomato-WPRE-bGH, AAV2/1-EF1a-fDIO-GCamP6s-wpre-sv40 and AAV-DJ CMV DIO eGFP-2A-TeNT. To achieve a sparse labeling of PNs for dendritic imaging experiments (Figures 3H and 3I) CAV2-Cre was injected into subcortical regions (amygdala and striatum) receiving inputs from ACx (injection coordinates: 1.7 mm posterior of bregma, 3.47 mm lateral of midline, 4-3.8 mm ventral). AAV2/1-CAG-flex-tdTomato-WPRE-bGH, AAV2/1-CAG-flex-GCaMP6s-WPRE-SV40 and AAV2/1-EF1a-fDIO-ChrimsonR-wpre-sv40 were co-injected in ACx of NDNF-Ires-FlpO mice.

A round cover glass (diameter 3 mm) glued to a section of hypodermic tubing (outer diameter 3 mm, 0.5 mm deep) was used to cover the craniotomy, and fixed using Cyanoacrylate glue (Ultra Gel, Henkel, Düsseldorf, Germany) and dental cement (Paladur, Heraeus, Hanau, Germany). The window was protected from dirt and light with silicone adhesive (Kwik-Cast).

Fear conditioning

Fear conditioning and fear retrieval took place in two different behavioral contexts (context A and B). The conditioning and test boxes and the floor were cleaned before and after each session with 70% ethanol or 1% acetic acid, respectively. CS for differential fear conditioning were 10 s (during conditioning) or 30 s (during recall) long trains of frequency-modulated sweeps (500 ms duration, logarithmically modulated between 5 and 20 kHz, 50 ms rise and fall) delivered at 1 Hz at a sound pressure level of 75 dB at the speaker (MF1 speakers and RZ6 processor, Tucker-Davis Technology). Up-sweep and down-sweep were used in a counterbalanced fashion as CS+. The CS+ was paired with a foot-shock (1 s, 0.6 mA, 15 CS+/foot-shock pairings; inter-trial interval: 20–180 s). The onset of the foot-shock coincided with the onset of the last sweep in the CS+. The CS- was presented after each CS+/foot-shock association, but was never reinforced (15 CS- presentations, inter-trial interval: 20–180 s). For pseudoconditioning the same sound stimuli were used. Sound stimuli (CS1 and CS2) and foot-shocks were presented separately in a random fashion (15 presentations each, inter-trial interval: 20–180 s). Conditioned mice were submitted to fear retrieval in context B, during which they received 4 presentations of CS^-(CS1) and CS+(CS2) trains. To score freezing behavior, we used a webcam (HD C270, Logitech) and custom written MATLAB (Mathworks) software (FreezingScoring). Mice were considered to be freezing if no movement was detected for 2 s and the measure was expressed as a percentage of time spent freezing. Animals with successful fear memory acquisition (learners) were defined by CS+ evoked freezing > 40% of the time during memory retrieval (n = 14, mean: 81%, range: 53 to 99%). Non-learners were defined as showing < 40% freezing during retrieval (n = 2, 36 and 18% CS+ freezing), which was similar to pseudoconditioned animals (n = 5, 24.6% mean CS freezing).

Slice preparation and whole-cell recordings

Animals expressed Channelrhodopsin-2 from AAV2/5.EF1a.DIO.hCHR2(H134R)-EYFP or AAV2/5.EF1a.dflox.hCHR2(H134R)-mCherry vectors for investigation of inhibitory input to NDNF-INs or output connectivity of NDNF and SST cells (300 nL virus, injected at from bregma: ap −2.46, lateral 4.45, depth from brain surface 0-0.6 mm). Excitatory input to NDNF cells was investigated by injecting 100 nL of AAV2/5.CamKIIa.hChR2(H134R)-EYFP into somatosensory cortex (S1, from bregma: ap −1.58, lateral 2.75, depth from brain surface 0-1.2 mm) or by injection of 250 nL of AAV2/5.EF1a.dflox.hCHR2(H134R)-mCherry into MGM (from bregma: ap −3.16, lateral 1.8, depth from brain surface 3.2 mm) in CR-Ires-Cre mice to target specifically the higher-order MGB whose projections are enriched in L1. Connectivity from NDNF to SST cells was assessed by injecting a mixture of AAV2/1-EF1a-fDIO-ChrimsonR (400 nl), AAV2/1-EF1a-fDIO-EYFP (150 nl) and AAV2/1.CAG.Flex.tdTomato (100 nl) to perform targeted recordings from SST cells. We note that while Flp-mediated expression was highly selective (Figures S1I and S1J), it reached slightly lower absolute expression levels than Cre-dependent constructs. Therefore, the more sensitive optogenetic effector Chrimson was chosen for these experiments.

For acute brain slices, animals were anesthetized with isoflurane (4%) in oxygen-enriched air (Oxymat 3, Weinmann, Hamburg, Germany), and decapitated into ice cold slicing solution containing (in mM): 93 NMDG, 93 HCl, 2.5 KCl, 1.2 NaH2PO4, 30 NaHCO3, 20 HEPES, 25 glucose, 5 sodium ascorbate, 2 thiourea, 3 sodium pyruvate, 10 MgSO4 and 0.5 CaCl2 (pH 7.3-7.4). Coronal slices (350μm thick) from auditory cortex (AuV/A1/AuD, located 2 to 3.4 mm posterior to bregma), were prepared on a vibratome (Leica VT 1200S), and transferred to an immersion style holding chamber filled with slicing solution at 34°C. After recovery for 15 minutes, slices were transferred to another holding chamber containing standard aCSF solution at RT containing (in mM): 125 NaCl, 3 KCl, 1.25 NaH2PO4, 26 NaHCO3, 10 glucose, 1 MgCl2 and 2 CaCl2 (pH 7.3-7.4) for 0.5−1 h before start of the recordings. All aCSF solutions were continuously bubbled with carbogen gas (95% O2, 5%CO2), and had an osmolality of 300 mOsm.

For recording, slices were transferred to the recording chamber and perfused with aCSF (2-3 mL/min). All experiments were performed at 31-34°C in the presence of DNQX (Sigma, 10 μM) and DL-AP5 (Biotrend, 25 μM) to avoid potential effects of recruiting PNs via disinhibition. Cells were visualized using differential interference contrast microscopy (Scientifica slice scope) and a water immersion objective (Olympus LUMPLFLN 40x, 0.8 N.A.). Fluorescently labeled neurons were visualized under epifluorescence using an LED (488 or 565 nm, Cool LED) and a CCD camera (Infinity3, Lumenera or Hamamatsu C11440 ORCA-flash4.0). Whole-cell voltage-clamp and current-clamp recordings were made using Multiclamp 700B amplifiers (Axon Instruments, CA), low-pass filtered at 5 to 10 kHz and digitized at 10 to 50 kHz (Digidata 1550, Molecular Devices) using pClamp software (Molecular Devices). Recordings were rejected or terminated when the access resistance exceeded 20 MΩ for PN recordings, and 25 MΩ for the smaller INs. Importantly, analyses indicate no difference in steady-state voltage clamp errors between experimental groups, and only a minor (< 1 ms) difference in voltage clamp time constant between experimental groups. Patch pipettes (4-6 MΩ) were pulled from standard-wall borosilicate capillaries and were filled with intracellular solution (in mm): 140 K-gluconate, 10 KCl, 10 HEPES, 4 Na-phosphocreatine, 4 ATP-Mg, 0.4 GTP and biocytin (4 mg/mL). pH was adjusted to 7.3 with KOH, and osmolality was 290-300 mOsm. Series resistance was left uncompensated, and values were not corrected for the liquid junction potentials. Steady-state and dynamic voltage clamp errors were calculated offline from the current response to a 10 mV hyperpolarizing pulse. Optogenetic stimulation was applied through the objective either full-field (standard), or exclusively to L1 (Figures 2E, 2F, and S3E–S3G) using an LED (488 nm, Cool LED). The LED pulse width was 0.5 ms and irradiance ranged from 1 to 45 mW/mm^2. IPSCs were evoked by trains of 4 pulses at 1Hz frequency for 10 consecutive sweeps (15 s inter-sweep interval), and recorded at a holding potential of −50 mV. For connectivity from NDNF to SST neurons (Figure 5D), and to neighboring PNs (Figure S6F), IPSCs were evoked with green light (0.5 ms, 532 nm, Cool LED). CGP-55845 (Sigma 3 μM), was bath applied. Long range input connectivity recordings (Figure 4L) were performed in tetrodotoxin (TTX, Alomone Labs, 1 uM) and 4-aminopyridine (4AP, Alomone Labs, 0.1-1 mM). Axons expressing ChR-2 in auditory cortex were stimulated with 488 nm pulses of 5 ms duration (62.7 mW/mm^2) every 15 s. Action potential bursts in critical frequency experiments (Figures 3A-3D and S4A–S4C) were evoked from resting membrane potential by brief current injection (3 pulses, 0.5 ms, 4 nA, tested frequency range was 25-125 Hz, assessed with increasing steps of 12.5 Hz with 10 s inter-stimulus-interval) and recorded with bridge balance compensation. To assess the effect of inhibition on dendritic spikes, L1 NDNF-INs were optogenetically stimulated with 4 pulses (0.5 ms) at 40 Hz frequency 100-50 ms before the last action potential in the burst. The effect of L1 NDNF-IN stimulation was tested in both the supra-critical frequency range (75 and 100 Hz), where a burst of three action potentials elicits a dendritic spike, and at sub-critical frequency (25 Hz) where a dendritic spike is never elicited. ADPs were quantified in Clampfit (Axon instruments) as the integral of a 40 ms window starting directly after the last action potential.

IPSC characteristics were determined from recordings with an amplitude of at least 10pA. First the average was generated from the 10 recorded trials and subsequently analyzed using custom MATLAB scripts. To quantify the onset of the IPSCs, traces were converted to z-scores and onset was defined as the first point that reaches 3 standard deviations above baseline. Rise time was calculated as the time for 20% to 80% of change between baseline and peak, and decay time as the 80%–20% fall time. To determine the charge, the integral of the IPSC was calculated in a temporal window of 800 ms.

In vivo *calcium imaging*

After 4 to 5 weeks for AAV expression and localization of auditory cortex by intrinsic imaging under anesthesia, animals were water restricted and habituated 3 times to handling and subsequently 3-4 times to head-fixation under the microscope, where they received water *ad libitum* before the experiment. For the fear conditioning experiments, in the habituation imaging session, each CS was presented 8 to 12 times. The CSs consisted of trains of 5 frequency-modulated sweeps (500 ms duration, logarithmically modulated between 5 and 20 kHz, 50 ms rise and fall) delivered at 1 Hz at a sound pressure level of 75 dB at the speaker (MF1 speakers and RZ6 processor, Tucker-Davis Technology). CS+ and CS- were presented in an alternating fashion. For the calculation of the mean response integral the first 4 stimulus presentations of CS- and CS+ during the habituation session were excluded to avoid effects of stimulus novelty. During the retrieval imaging session 24 h after fear conditioning (and 48 h after the habituation imaging session), the same neurons were imaged again and 16 CSs were presented (CS- and CS+ alternating, 8 each). In a subset of the data an additional retrieval imaging session was performed 2.5-3.5 h after the fear conditioning (data not shown). For sound intensity experiments, mice were presented with 5 white noise bursts (100 ms duration, 10 ms rise/fall, delivered at 5 Hz) at 60 dB, 75 dB, 90 dB and 105 dB (measured at the speaker) in a pseudorandom manner, with 7 presentations in total per intensity level. NDNF neurons and SST axons were recorded at a depth of 40- 80 μm and SST somata at a depth of 160-200 μm below the pia.

For the NDNF activation experiment (Figures 3E–3I), NDNF neurons expressing Chrimson were optogenetically activated using an orange LED (594 nm). A 500 ms light pulse (26.5 mW) was applied, immediately followed by 5 white noise bursts (100 ms duration, 10 ms rise/fall, delivered at 5 Hz) at 75 dB to evoke dendritic activity. Apical tuft dendrites were recorded simultaneously at a depth of 30-70 μm below the pia in L1. While it was not possible to determine from how many PNs the recorded dendrites originated in these experiments, based on post hoc analyses we estimate that we recorded from at least 60 independent PNs. In addition, even distal dendrites of the same PN can function as independent units *in vivo* (Major et al., 2013; Stuart and Spruston, 2015; Cichon and Gan, 2015). Thus, we treated each dendritic segment as independent in this analysis. LED-on and LED-off trials were presented in an alternating fashion (6-8 trials per field of view). Dendrites were defined as responsive to the noise stimulation if the mean z-score over all LED-off trials during the stimulation and 1 s after crossed a threshold of 1.29. For the control experiment in Figures 3F, 3G, and S4D–S4F, GCaMP6s was expressed together with Chrimson or alone in NDNF neurons, and changes in fluorescence were measured in response to light stimulation under both conditions.

Calcium imaging was performed with a resonant scanner microscope (Bruker Investigator equipped with single photon stimulation by a 594 nm LED, or custom built) and a femtosecond laser (Spectra Physics MaiTai or InSight) at 920 nm. The average excitation power under the objective (Nikon 16x, 0.8 N.A., 3 mm WD) was below 30 mW. Images (416x416 or 512x512 pixels) were acquired at 19 to 30 Hz. The imaging field of view was chosen at depths 15-75 μm below dura. Image acquisition, CS delivery and camera for pupil tracking (see below) were controlled using custom written software (AudioGame). Post hoc processing of the acquired time series consisted of motion correction using custom MATLAB code and 2-4x temporal binning resulting in a frame rate of approximately 5 Hz. The mean projection of the red channel was used to outline the regions of interest (ROIs) using ImageJ (Fiji) or a MATLAB based custom-written software. Fluorescent values were extracted from the image stacks for each ROI, and df/f was calculated as $(F-F_0)/ F_0 \times 100$, where F_0 is the mean fluorescence during < 15 s of the trial before stimulus onset. The response integral was calculated using trapezoidal numerical integration of the df/f trace during the stimulus presentation. Strongly responsive neurons were chosen based on a combination of two criteria: 1. The mean z-score of all trials of one session crossed a threshold of 1.96 during the stimulus presentation, and 2. In at least two trials of one session the z-score was > 1.96 for > 1 s during stimulus presentation. To quantify the response onset in the sound intensity experiments we extracted all responsive trials of all ROIs across all four sound intensities based on a significant z-score (> 1.96) during the stimulus window. We then calculated the time point when the response reached 10% of the maximum peak during the stimulus window. For this analysis we used the data without prior binning (frame rate: ~30 Hz).

Measurement of pupil diameter

Recording of pupil diameter was performed using a camera (Basler acA1920-25um) and custom-written software (EyeTracker) at a frame rate of approximately 20 Hz under infrared illumination (LED, λ = 620nm). Data was binned in the time domain to reach a sampling rate of approximately 5 Hz. The change in pupil diameter ($\Delta d/d$) was calculated as $(d-d_0)/ d_0 \times 100$, where d_0 is the mean diameter during the first < 15 s of each trial before stimulus onset. The response integral was calculated using trapezoidal numerical integration of the $\Delta d/d$ trace during the 10 s following stimulus onset.

QUANTIFICATION AND STATISTICAL ANALYSIS

The number of experimental recordings and animals used in each experiment is indicated in the figure legends. Statistical tests were performed using GraphPad Prism and MATLAB, p values and statistical tests used are indicated in the figure legends. Data were first subjected to a Shapiro-Wilk test of normality, and based on the result to the indicated parametric and non-parametric tests.

DATA AND SOFTWARE AVAILABILITY

The custom-written software used for data acquisition and analysis is available under the following links:

AudioGame

Synchronization of sound presentation, pupil tracking and 2-photon imaging
https://software.scic.brain.mpg.de/projects/MPIBR/AudioGameGUI/

EyeTracker and Camera Acquisition

Recording of pupil videos and tracking of pupil dilation
https://software.scic.brain.mpg.de/projects/PylonRecorder/PylonRecorder
https://software.scic.brain.mpg.de/projects/PylonRecorder/TrackerPlugin_EyeTracker

Processing of calcium imaging data

Motion correction of Calcium imaging data
https://software.scic.brain.mpg.de/projects/MPIBR/CellSortPCAICA

FreezingScoring

https://github.molgen.mpg.de/MPIBR/FreezingAnalysis

Neuron, Volume *100*

Supplemental Information

Learning-Related Plasticity

in Dendrite-Targeting Layer 1 Interneurons

Elisabeth Abs, Rogier B. Poorthuis, Daniella Apelblat, Karzan Muhammad, M. Belen Pardi, Leona Enke, Dahlia Kushinsky, De-Lin Pu, Max Ferdinand Eizinger, Karl-Klaus Conzelmann, Ivo Spiegel, and Johannes J. Letzkus

Supplemental Figures

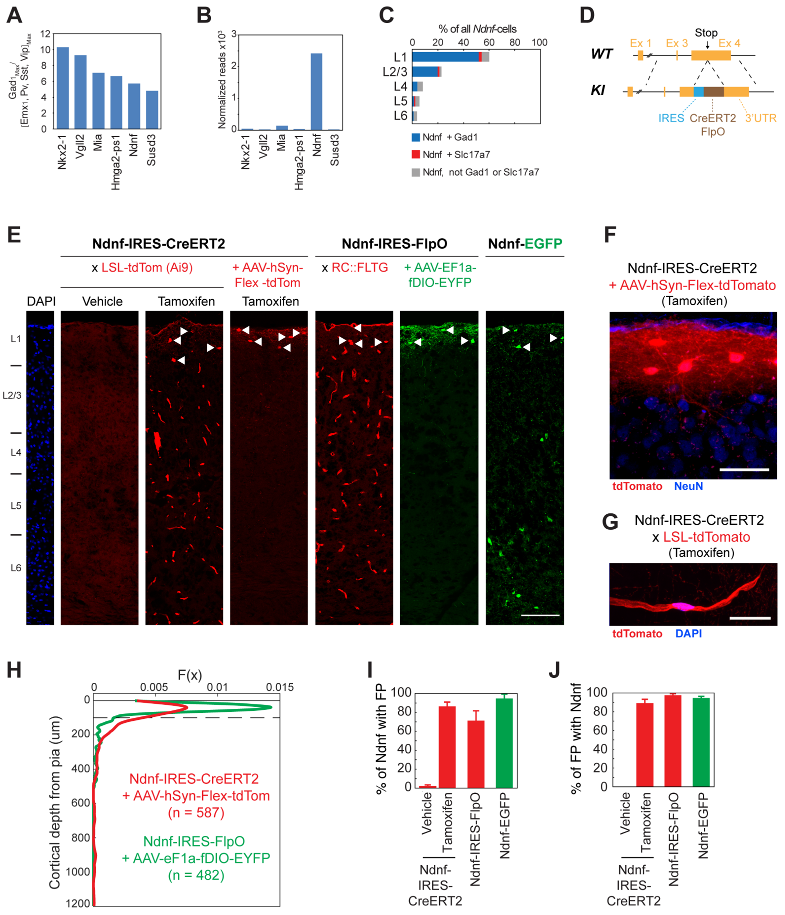

Supplemental Figure 1: Identification of Ndnf as selective marker for L1 INs and generation of mouse lines for selective labeling of* L1 NDNF-INs*. Related to Figure 1

A Enrichment score of the six genes that were identified in the RiboTag-Seq data as highly enriched in Gad2-neurons as compared to excitatory neurons and PV-, SST- and VIP-INs ($Gad2_{Max}/[Emx1/Pv/Sst/Vip]_{Max} \geq 4$). **B** Expression levels of these genes in the Gad2-Cre line as measured by RiboTag-Seq (normalized reads x10^3). **C** Quantification of triple FISH for *Ndnf*, *Gad1* and *Slc17a7* (= *VGlut1*) in the adult auditory cortex. Note that *Ndnf*-expressing cells in lower cortical layers are mainly non-neuronal (i.e. *Gad1* and *Slc17a7* negative). **D** Targeting strategy for generation of Ndnf-Ires-CreERT2 and Ndnf-Ires-FlpO mice. A cassette containing Ires-CreERT2 or Ires-FlpO was introduced by homologous recombination into the 3' UTR of the *Ndnf*-gene in Exon 4 after the Stop codon. **E** Reporter expression in Ndnf-Ires-CreERT2 mice, Ndnf-Ires-FlpO mice and in transgenic Ndnf-EGFP mice (Gong et al., 2003). Ndnf-Ires-CreERT2 mice were crossed to mice that express tdTomato upon Cre expression (Ai9, Madisen et al., 2010). Cre activity in the double-heterozygous offspring is observed only upon application of tamoxifen, and reporter expression is observed in L1-INs (arrowheads) as well as in blood vessels throughout all cortical layers (see **G**). Selective labeling of L1 NDNF-

INs (arrowheads) without reporter expression in blood vessels is achieved by injection of Cre-dependent AAVs into the cortex of Ndnf-Ires-CreERT2 mice and subsequent tamoxifen injection (see also **F** for high magnification). Analogous experiments on Ndnf-Ires-FlpO mice. Ndnf-Ires-FlpO mice were crossed to mice that express tdTomato upon Flp expression (RC::FLTG), revealing expression in both blood vessels and L1-INs (arrowheads). Injection of Flp-dependent AAVs into the cortex of these mice labels selectively L1 NDNF-INs (arrowheads). Ndnf-EGFP labels L1 NDNF-INs (arrowheads) and PNs in lower cortical layers (no labeling of PNs in any layers was observed in Ndnf-Ires-CreERT2 mice) (scale-bar in **E** 200 µm, in **F, G** 20 µm). **H** Distribution of tdTomato- or EYFP-labeled cells in Ndnf-Ires-CreERT2 and Ndnf-Ires-FlpO mice injected with conditional AAVs (AAV-hSyn-Flex-tdTomato or AAV-EF1a-fDIO-EYFP). Labeled cells are highly concentrated in L1 in both lines. **I, J** *Ndnf*-expression in L1 is accurately reported by Ndnf-Ires-CreERT2, Ndnf-Ires-FlpO and Ndnf-EGFP mice. FISH for *Ndnf* and the respective fluorescent protein (*tdTomato* or *Egfp)* was done in adult brains of either double heterozygous Ndnf-Ires-CreERT2 x Ai9 mice or of Ndnf-EGFP mice. The percentages of co-expressing cells in L1 of the auditory cortex are plotted in the bar graphs. Error bars are s.e.m.

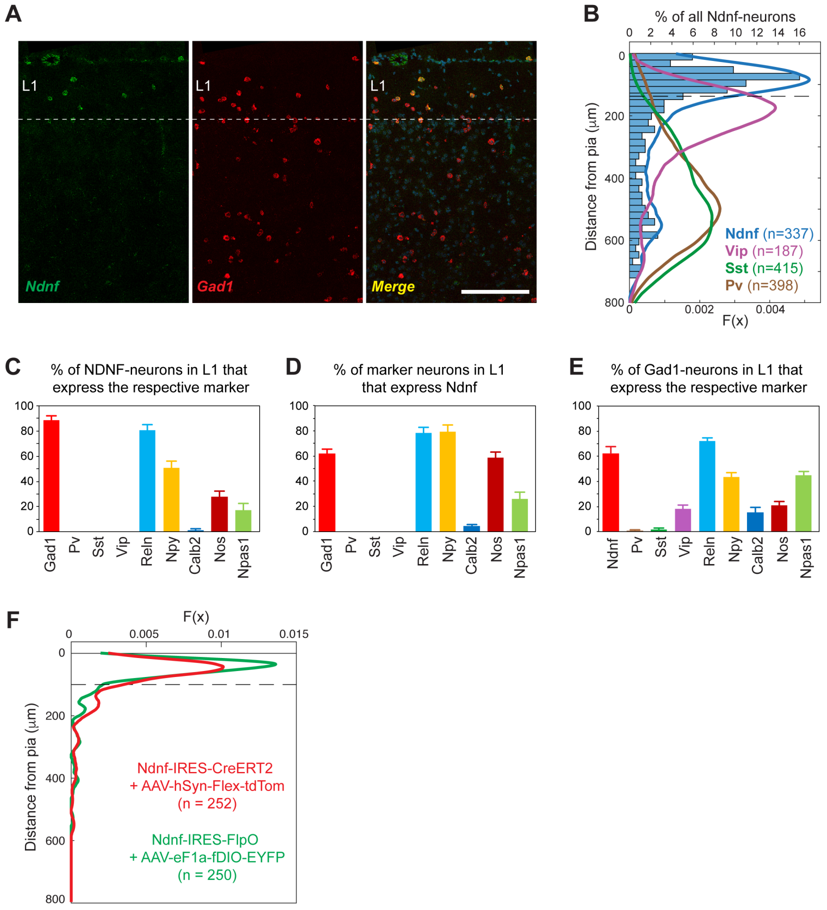

Supplemental Figure 2: Ndnf as selective marker for L1 INs in prefrontal cortex and targeting by novel mouse lines. Related to Figure 1

A Representative image of FISH for *Ndnf* and *Gad1* in adult prefrontal cortex (DAPI-labeled nuclei are in blue, scale-bar = 200 µm). **B** The distribution of *Ndnf*-expressing INs differs from the distribution of other INs in prefrontal cortex. The distance from the pia was determined for each cell expressing a given marker, and plotted as a histogram (for *Ndnf*) or the corresponding Probability Density Function (PDF, for *Ndnf*, *Pv*, *Sst* and *Vip*, dashed line indicates the L1 border). **C-F** *Ndnf*-expressing neurons constitute the majority of L1 GABAergic neurons and do not overlap with *Pv*, *Sst* or *Vip*. **C** Percentage of L1 *Ndnf* neurons that co-express the respective marker. **D** Percentage of L1 neurons that express the respective subtype marker and that co-express *Ndnf*. **E** Percentage of Gad1-positive L1 neurons that express each of the subtype markers. **F** Two newly generated mouse lines allow for selective labeling of L1 NDNF neurons in prefrontal cortex. Distribution of tdTomato- or EYFP-labeled cells in Ndnf-Ires-CreERT2 and Ndnf-Ires-FlpO mice injected with an conditional AAVs (AAV-hSyn-Flex-tdTomato or AAV-EF1a-fDIO-EYFP, respectively). Labeled cells are highly concentrated in L1 in both lines. Data are presented as mean ± s.e.m.

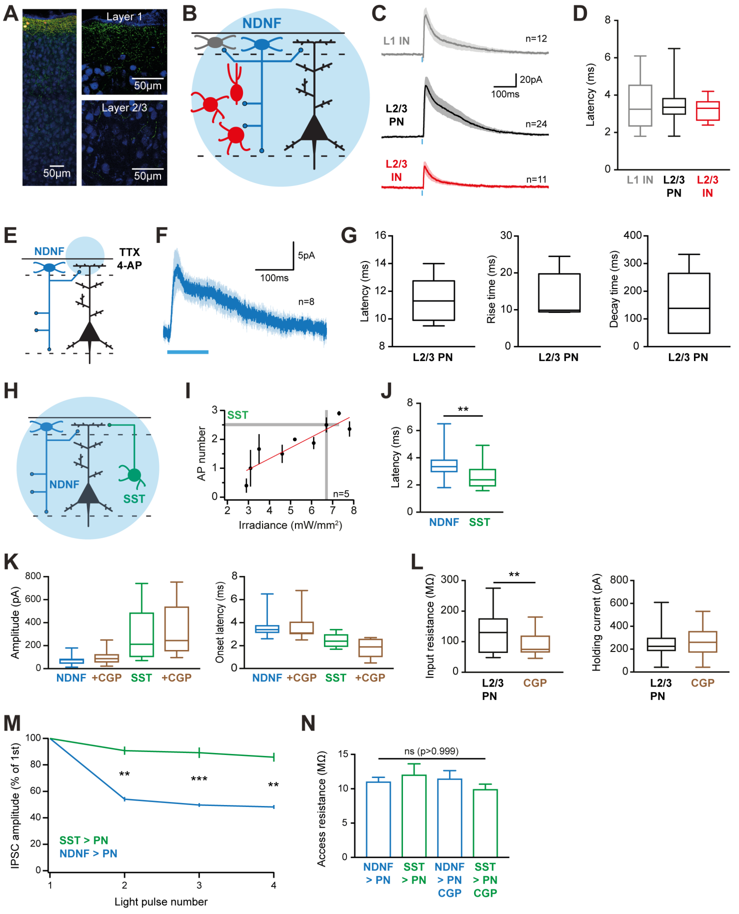

Supplemental Figure 3: Further characterization of output connectivity of layer 1 NDNF interneurons in auditory cortex. Related to Figure 2

A Overview (**left**) and high magnification images (**right**) of L1 NDNF-IN output synapse location after AAV-mediated expression of cytosolic tdTomato (red) and synaptophysin-GFP fusion protein (green) in auditory cortex of Ndnf-Ires-CreERT2 mouse line counterstained for NeuN (blue). Note the higher density of GFP puncta in L1 compared to L2/3 (images on right are projections over 2 µm imaging depth). **B, C** Schematic of optogenetic mapping and average traces of optogenetically-evoked IPSCs in CHR-2 negative L1-INs (grey, n=12), L2/3 PNs (black, n=24) and L2/3 INs (red, n=11). **D** Optogenetically-evoked IPSCs showed short onset latencies that did not differ between the postsynaptic populations (Kruskal-Wallis H-test with Dunn's multiple comparison). **E, F** Schematic and average IPSC in response to optogenetic activation of L1 NDNF-IN synapses selectively in L1 under action potential block (TTX, 1 µM, 4-AP 100 µM). **G** Latencies, rise and decay times of IPSCs in these experiments (n=8). **H** Schematic of experiments comparing the properties of NDNF- and SST-IN input to the distal dendrites of L2/3 PNs. **I** Calibration of optogenetic stimulation of ChR-2 expressing SST-INs. Across the population (n=5), the chosen irradiance (grey lines, 6.8 mW/mm^2) elicited 2.5 action potentials per pulse (0.5 ms). **J** The onset latency of L1 NDNF-IN input to

L2/3 PNs was significantly longer than for SST input, consistent with the slower rise and decay times of this input (**Figure 2I**, Mann-Whitney test). **K** Bath application of the selective $GABA_B$ receptor antagonist CGP 55845 (3 μM) caused no significant change in amplitude or onset latency of IPSCs from L1 NDNF-INs (n=9) or SST-INs (n=7, Wilcoxon test within NDNF and SST groups). **L** CGP 55845 (3 μM) caused only modest effects on the intrinsic properties of L2/3 PNs (n=16, Wilcoxon test). **M** Short-term dynamics of SST- and NDNF-IN input to PNs during optogenetic train stimulation normalized to the amplitude of the first IPSC. Note stronger short-term depression of L1 NDNF-IN output synapse, consistent with previous observations during paired recordings (Capogna and Pearce, 2011; Olah et al., 2009; Overstreet-Wadiche and McBain, 2015; Tamas et al., 2003). **N** Maximal access resistance of recordings from L2/3 PNs during input from L1 NDNF-INs (n=24) or SST-INs (n=13), and of the subset of these recordings in which the $GABA_B$ receptor antagonist CGP 55845 was applied (L1 NDNF-INs n=9, SST-INs n=7). There is no difference in access resistance between these groups (Kruskal-Wallis H-test with Dunn's multiple comparison, p>0.999), indicating that recording quality cannot explain the physiological differences we observe. C, F, I, N show mean ± s.e.m., other plots range, quartiles and median.

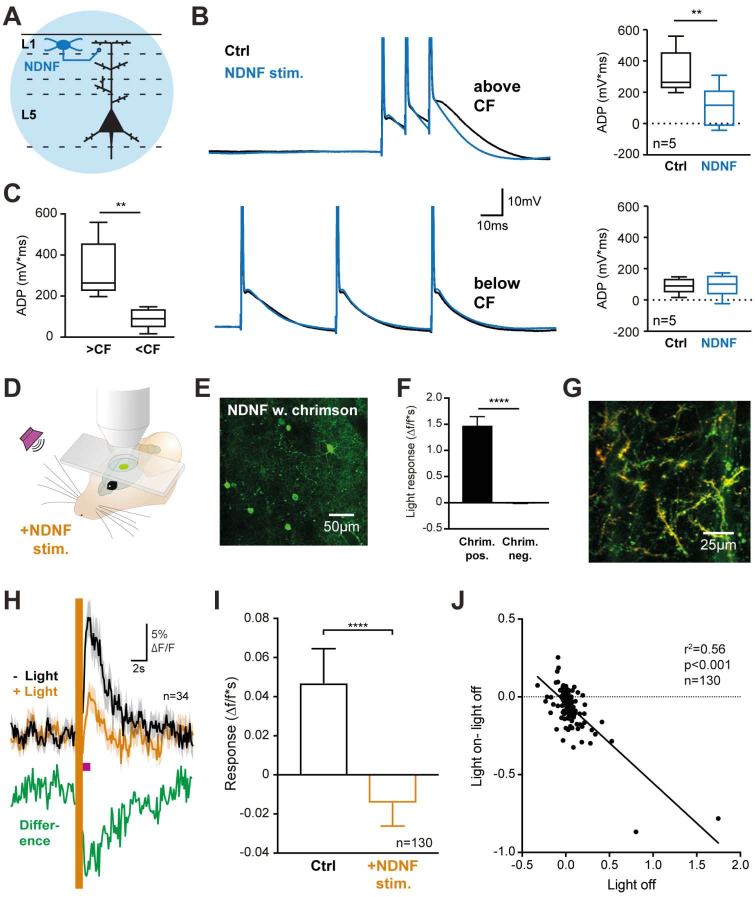

Supplemental Figure 4: Further characterization of pyramidal neuron dendritic activity control by layer 1 NDNF interneurons. Related to Figure 3

A Schematic of critical frequency experiment in L5 PNs. **B** Example recording (left) and quantification (right, n=5) showing the selective reduction of the afterdepolarization (ADP) after an action potential burst by preceding L1 NDNF-IN stimulation (4 pulses at 40Hz, ending 50-100 ms before last action potential) for supra- but not sub-critical frequency stimulation (paired t-test). **C** The ADP after a burst of 3 action potentials was greater for supra- than for sub-critical frequency stimulation (n=9, paired t-test). **D** *In vivo* 2-photon imaging in auditory cortex of awake mice combined with sensory stimulation (magenta, 5 white noise bursts, 100 ms duration, delivered at 5 Hz) and optogenetic activation of L1 NDNF-INs (yellow, 594 nm). **E** Field of view during *in vivo* imaging of L1 NDNF-INs co-expressing GCaMP6s (green) and the optogenetic effector Chrimson in Ndnf-Ires-FlpO mice. **F** Optogenetic activation elicited strong responses in L1 NDNF-INs expressing Chrimson (n=78), and no activity in animals that only expressed GCaMP6s (n=134, Mann-Whitney test). **G** Field of view during *in vivo* imaging of distal PN dendrites in L1 expressing GCaMP6s (green) and tdTomato (red) used for motion correction. PNs were selectively labeled by a combination of retrograde Cre-expression from subcortical regions and Cre-dependent expression of GCaMP6s and tdTomato in auditory cortex. **H** Sensory responses (black) in dendritic branches that displayed

significant activation by auditory stimulation (34 dendrites in 3 mice, see Methods for details). Optogenetic activation of L1 NDNF-INs (yellow) immediately preceding auditory stimulation (magenta) caused a long lasting reduction of dendritic responses (difference trace in green). **I** There was a highly significant reduction in dendritic responses also when both dendrites responsive and unresponsive to sensory stimulation were considered (130 dendrites in 3 mice, Wilcoxon test, see Methods for details). **J** There was a highly significant correlation between the size of sensory responses in a dendrite (light off) and the effect of L1 NDNF-IN activation (light on – light off, r^2=0.56, p<0.001, n=130), revealing that stronger sensory responses were suppressed more. F, H, I shows mean ± s.e.m., other plots range, quartiles and median.

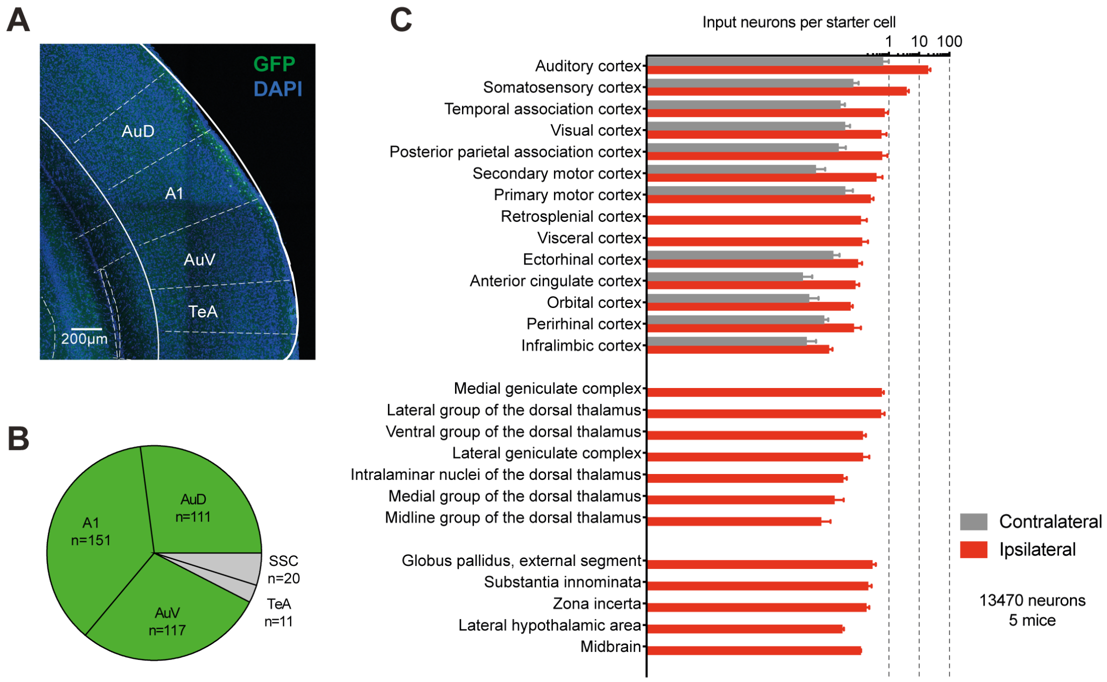

Supplemental Figure 5: Further characterization brain-wide sources of synaptic input to auditory cortex layer 1 NDNF interneurons. Related to Figure 4

A Representative image after injection of AAV-synP-DIO-sTpEpB (Kohara et al., 2014) to make L1 NDNF-INs competent for transduction by rabies virus. Note expression in auditory cortex layer 1. The mCherry channel revealing presynaptic input neurons is omitted here for clarity, see **Figure 4A** for a two color image. **B** Area localization of all starter L1 NDNF-INs identified by GFP and mCherry co-localization (410 cells, 5 mice). 92% of starter cells were located in auditory cortex (A1 primary auditory cortex, AuD dorsal auditory cortex, AuV ventral auditory cortex). An additional 3% was located in temporal association cortex (TeA), whereas 5% were found in adjacent somatosensory cortex (SSC). **C** The same data as in **Figure 4C** is presented here by the complementary measure of number of input neurons per starter cell. Plot shows mean ± s.e.m.

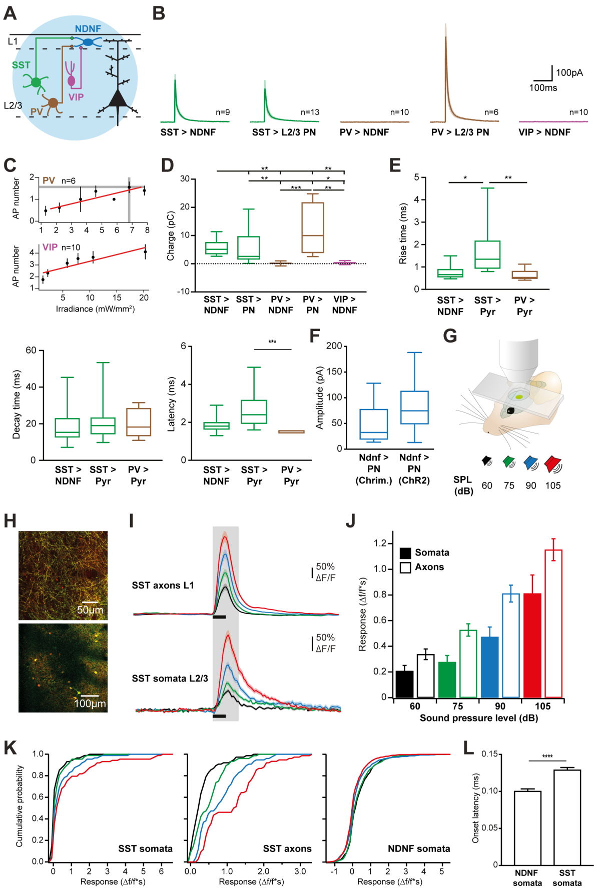

Supplemental Figure 6: Further characterization of inhibitory control of layer 1 NDNF interneuron activity in auditory cortex. Related to Figure 5

A, B Schematic and average traces of optogenetically-evoked IPSCs in L1 NDNF-INs (from SST n=9, from PV n=10, from VIP n=10) and L2/3 PNs recorded for comparison (from SST n=13, from PV n=6). The connection from VIP-INs to PNs was not tested due to the weak connectivity observed in previous studies (Pfeffer et al., 2013; Pi et al., 2013). **C** Calibration of optogenetic stimulation of ChR-2 expressing PV- and VIP-INs. In PV-INs (n=6), the chosen irradiance (grey lines, 6.8 mW/mm^2) elicited 1.6 action potentials per pulse (0.5 ms). Connections from VIP-INs were tested at different irradiance levels between 5 and 20 W/mm^2, which elicit robust action potential firing (n=10). **D** L1 NDNF-INs receive strong inhibition from SST-INs similar to L2/3 PNs, but no input from PV- or VIP-INs (Kruskal-Wallis H-test with Dunn's multiple comparison). **E** While decay times were similar in the different experiments, rise times of SST input to L2/3 PNs were longer than in the other populations, consistent with the distal dendritic location of these synapses. In addition, the onset latencies of PV-IN input to PNs was faster than in the other populations, consistent with the perisomatic localization and rapid kinetics of these synapses (Kruskal-Wallis H-test with Dunn's multiple comparison). **F** Comparison of IPSC amplitudes elicited in PNs by optogenetic stimulation after Cre-dependent expression of ChR-2 and Flp-dependent expression of Chrimson. No

difference was observed ($p>0.05$, unpaired t-test), indicating similar efficiency of L1 NDNF-IN stimulation in both experiments. **G** Schematic for *in vivo* 2-photon imaging in auditory cortex of awake mice during stimulation with white noise bursts (5 bursts, 100 ms duration, delivered at 5 Hz) at different sound pressure levels. **H** Fields of view during *in vivo* imaging of SST axons in L1 (**top**), SST-INs in L2/3 (**bottom**) in auditory cortex. **I** SST-IN axons (**top**, n=11 regions in 11 mice) and somata (**bottom**, n=88 in 5 mice) displayed similar response increases with increasing stimulus intensity (60-105 dB SPL, color code in **G**). **J** Statistics of responses in **I** (Kruskal-Wallis test with Dunn's multiple comparison, only significant differences are reported, somata: s, axons: a: s 60dB vs s 90dB: $p<0.05$, s 60dB vs s 105dB: $p<0.0001$, s 60dB vs a 60dB: $p<0.01$, s 60dB vs a 75dB: $p<0.0001$, s 60dB vs a 90dB: $p<0.0001$, s 60dB vs a 105dB: $p<0.0001$, s 75dB vs s 105dB: $p<0.05$, s 75dB vs a 75dB: $p<0.0001$, s 75dB vs a 75dB: $p<0.0001$, s 75dB vs a 90dB: $p<0.0001$, s 75dB vs a 105dB: $p<0.0001$, s 90dB vs a 90dB: $p<0.0001$, s 90dB vs a 105dB: $p<0.0001$, s 105dB vs a 90dB: $p<0.001$, s 105dB vs a 90dB: $p<0.001$, s 105dB vs a 105dB: $p<0.0001$, a 60dB vs a 90dB: $p<0.0001$). This indicates that both approaches yield comparable results, and that imaging SST axons is a valid approach to determine the inhibition that arrives in L1 from this source (c.f. Lovett-Barron et al., 2014). **K** Cumulative histograms of response integrals of SST somata in L2/3 (left, 88 neurons per SPL, 5 mice), SST axons in L1 (center, 77 responses per SPL, 11 regions in 11 mice) and L1 NDNF-INs (right, 95 neurons per SPL, 5 mice). Note opposite effects of stimulus intensity across the entire population. **L** Latencies of the initial excitatory component in L1 NDNF-INs and SST axons. Note faster onset of L1 NDNF-IN excitation (Mann-Whitney test). B, C, I, J, L show mean ± s.e.m., other plots range, quartiles and median.

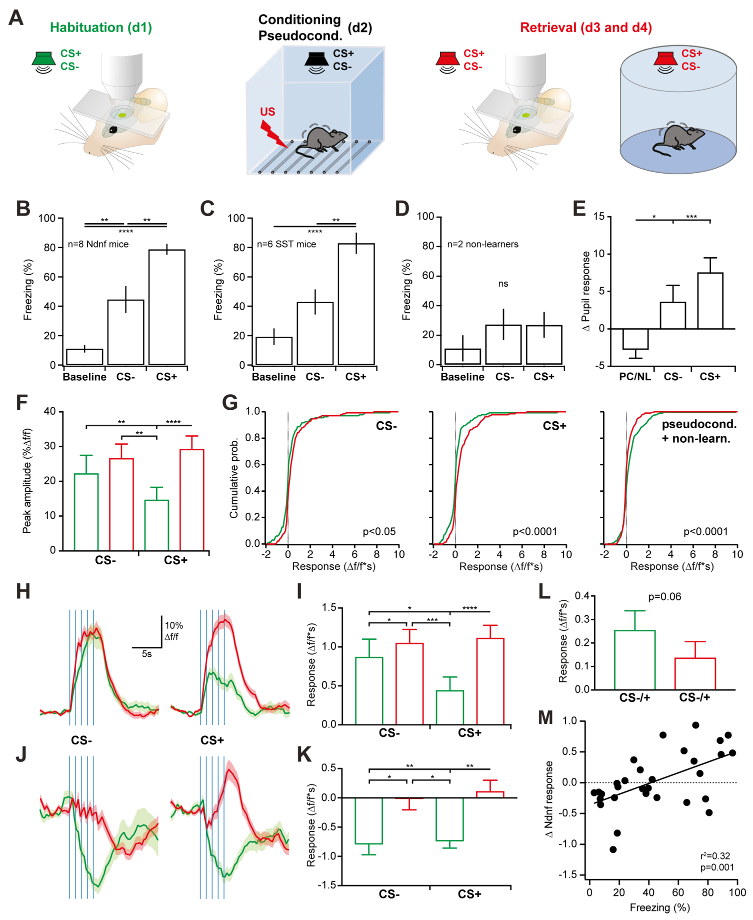

Supplemental Figure 7: Further characterization of plasticity of layer 1 NDNF interneuron stimulus responses after associative learning. Related to Figure 6

A Experimental schematic. **B, C** Freezing behavior of fear conditioned NDNF (**B**, n=8, CS+: 4 up sweeps, 4 down sweeps) and SST animals (**C**, n=6, CS+: 4 up sweeps, 2 down sweeps) in a freely-behaving memory retrieval session on day 3 or 4 indicates strong, discriminative fear memory (one-way ANOVA NDNF $F(2, 21)=32.7$, $p<0.0001$; one-way ANOVA SST $F(2, 15)=19.8$, $p<0.0001$ Tukey's multiple comparison test). **D** Freezing behavior of 2 fear conditioned NDNF animals which failed to form a stable memory (criterion: CS+ freezing < 40%, one-way ANOVA NDNF $F(2, 3)=0.97$, $p>0.05$). **E** Change in CS-evoked pupil responses between the first and second imaging session for pseudoconditioning and non-learners (CS1 and 2 combined, 7 mice) and fear conditioning (8 mice, One-way ANOVA $F(2, 27)=12.2$, $p<0.001$, Tukey's posthoc test). **F** Similar to the response integral (**Figure 6H**), the peak amplitudes of L1 NDNF-IN responses were similar during habituation ($p=0.85$, green), and strongly increased for the CS+ during retrieval (red), with a modest increase also observed for the CS- (133 neurons in 8 mice, CS+: 58 neurons up sweeps, 75 neurons down sweeps, Friedman test with Dunn's multiple comparison). **G** Cumulative histograms of response integrals for CS-, CS+ and pseudoconditioned stimuli (pseudoconditioned and non-learners: 128 neurons in 7 mice, both CSs combined). Note the potentiation of CS+ responses across

the population (Wilcoxon test). **H** Average timecourse of CS responses of strongly activated L1 NDNF-INs (see Methods, CS+ 69 neurons, CS- 68 neurons) showing a modest increase for the CS-, and strong potentiation of CS+ responses (onset of each sweep marked by blue lines). **I** Quantification of the response integral for data in **H** (Friedman test with Dunn's multiple comparison). Both CS- and CS+ responses were significantly potentiated after fear conditioning, whereas no difference was observed during habituation. **J** Same as **H** for strongly inhibited L1 NDNF-INs (see Methods, CS+: 20 neurons, CS-: 16 neurons, Friedman test with Dunn's multiple comparison). Inhibitory CS responses are reduced for the CS-, and converted to delayed excitation for the CS+. **K** Quantification of the response integral for data in **J**. **L** Response integral from 2 fear conditioned NDNF animals which failed to form a stable memory (n=44, Wilcoxon test). Since no difference in behavior was observed (**D**), responses to both CSs are presented combined. **M** Correlation between the response change in L1 NDNF-INs due to fear conditioning (response integral retrieval minus integral habituation) and freezing behavior elicited by that stimulus for fear conditioned and pseudoconditioned animals indicates that potentiation of L1 NDNF-INs correlates with learned stimulus relevance. Data are presented as mean ± s.e.m.
